# The pathogenesis of benign prostatic hyperplasia and the roles of Prdx3, oxidative stress, pyroptosis and autophagy:a review

**DOI:** 10.3389/fonc.2025.1579539

**Published:** 2025-08-05

**Authors:** Junjie Xiang, Yuxuan Zheng, Diang Chen, Yining Zeng, Jingqi Zhang, Degui Chang, Cheng Chang

**Affiliations:** ^1^ Department of Urology, TCM Prevention and Treatment of Metabolic and Chronic Diseases Key Laboratory of Sichuan Province, Hospital of Chengdu University of Traditional Chinese Medicine, Chengdu, China; ^2^ Acupuncture and Tuina School, Chengdu University of Traditional Chinese Medicine, Chengdu, China; ^3^ Eye School of Chengdu University, Traditional Chinese Medicine, Chengdu, China; ^4^ Jiangsu Provincial Hospital of Chinese medicine, Affiliated Hospital of Nanjing University of Chinese Medicine, Nanjing, Jiangsu, China

**Keywords:** benign prostatic hyperplasia, hormone, inflammation, peroxiredoxin 3, pyroptosis, autophagy, oxidative stress

## Abstract

Benign prostatic hyperplasia (BPH) is driven by hormonal and inflammatory mechanisms, yet emerging factors such as peroxiredoxin 3 (Prdx3), oxidative stress (OS), pyroptosis, and autophagy remain understudied. This review synthesizes their roles in BPH pathogenesis. We demonstrate that Prdx3 inhibits autophagy, exacerbates OS, and induces pyroptosis, ultimately promoting prostate cell proliferation. Paradoxically, while Prdx3 mitigates OS, its interaction with autophagy amplifies oxidative damage. These findings challenge conventional antioxidant therapies, suggesting that enhancing antioxidant capacity may inadvertently worsen BPH progression. Our analysis provides novel insights into therapeutic strategies targeting these pathways.

## Introduction

Benign prostatic hyperplasia (BPH) is a prevalent age-related urological disorder affecting men globally. Epidemiologically, the risk of developing histological BPH approaches 100% in males surviving beyond age 80 (1). However, persistent ambiguity in BPH terminology frequently results in clinical mismanagement, including overtreatment of subclinical cases or undertreatment of symptomatic presentations mimicking BPH (2). Consequently, establishing precise diagnostic criteria is essential for both research and clinical practice.

Histologically, BPH is defined as benign proliferation of stromal and glandular epithelial cells within the prostatic transition zone, accompanied by hyperplasia of smooth muscle and connective tissue—collectively forming a benign adenoma (3). Progressive enlargement (benign prostatic enlargement, BPE) may cause bladder outlet obstruction (BOO) through mechanical compression and increased glandular resistance, leading to lower urinary tract symptoms (LUTS) (3). Notably, BOO and LUTS are not pathognomonic for BPH (4), as their clinical significance depends more on anatomical location than absolute prostate size—a key factor contributing to therapeutic inaccuracy.

LUTS substantially impair quality of life through two symptom domains: obstructive manifestations (hesitancy, weak stream, straining, prolonged voiding) and irritative symptoms (frequency, urgency, nocturia, incomplete emptying, dribbling, incontinence, acute retention) (1, 5).

The clinical diagnosis of BPH therefore requires:(1)Histologically confirmed benign adenoma;(2)Documentation of LUTS attributable to prostatic pathology;(3)Exclusion of alternative causes (6).

Diagnostic evaluation integrates symptom assessment (IPSS questionnaire), digital rectal examination, urinalysis, post-void residual volume measurement, transrectal ultrasonography (measuring intravesical protrusion and prostate volume), serum PSA, and renal function tests (7, 8). Standardized application of these criteria is fundamental for accurate phenotyping in research and targeted clinical management.The comprehensive diagnostic criteria and symptom profiles are summarized in **Table 1**.

**Table 1:** Definition and diagnostic evaluation of BPH.

Category	Definition/Diagnostic criteria
Histological Definition	Benign proliferation of stromal and glandular epithelial cells within the prostatic transition zone, forming a non-malignant adenoma.
Clinical Definition	Diagnosis requires all three criteria: 1. Histologically confirmed benign adenoma 2. Documented lower urinary tract symptoms (LUTS) attributable to prostatic pathology 3. Exclusion of alternative etiologies
Symptom Profile	Obstructive symptoms:1.Hesitancy 2.Weak urinary stream 3.Straining 4.Prolonged voiding Irritative symptoms:1.Urinary frequency 2.Urgency 3.Nocturia 4.Incomplete bladder emptying 5.Post-void dribbling 6.Urge incontinence 7.Acute urinary retention
Diagnostic Workup	Combining symptoms, imaging examinations, digital rectal examination, urinalysis, residual urine, bladder protrusion of the prostate, prostate volume, Prostate-Specific Antigen, creatinine, etc., for a comprehensive evaluation to answer the question.

## Pathogenesis of BPH

The current understanding of BPH pathogenesis attributes a central role to hormones and inflammation. Emerging evidence also implicates factors such as Peroxiredoxin 3 (Prdx3), autophagy, oxidative stress(OS), and pyroptosis as potential contributors to BPH onset.

### Hormones

The occurrence and progression of BPH are closely linked to hormonal factors, predominantly sex hormones, thyroid hormones, insulin-like growth factor 1 (IGF-1), and insulin (9).

### Sex hormones

Sex hormones associated with BPH primarily involve androgens and estrogens. Additionally, progestogens are relevant, as progesterone receptor expression correlates positively with BPH development (10).

Androgens constitute established risk factors for BPH. The prostate, being hormone-sensitive, requires androgens and androgen receptors (AR) for tissue growth and development. Thus, discussion of BPH cannot disregard androgens and AR.

Dihydrotestosterone (DHT)—the active metabolite of testosterone—is synthesized by 5α-reductase (11).

Two isoforms of 5α-reductase (type 1 and 2) exist; clinically, finasteride (a type 2 inhibitor) blocks testosterone conversion to DHT, thereby treating BPH. However, such drugs may increase venous thromboembolism (VTE) risk (12).

DHT activates AR, driving prostate cell proliferation and resultant hyperplasia.Studies by Jin-Wen Kang et al. demonstrated that enhanced AR sensitivity via the Notch1 signaling pathway promotes BPH development, indicating that androgen receptor activation alone can drive prostate cell proliferation (13). Woo Yong Park et al. reported that AR inhibition with ellagic acid effectively treats BPH (14). Additional studies revealed that downregulating the PlncRNA-AR/androgen axis through the PI3K/AKT pathway delays prostatic hyperplasia (15).

In summary, androgens and AR-related factors significantly contribute to BPH pathogenesis, with some scholars considering them decisive factors in BPH development. However, evidence suggests that BPH may not develop despite androgen excess and can occur under androgen-deficient conditions (16).

A significant proportion of BPH cases are androgen-independent, and BPH typically develops as serum testosterone levels decline after the plateau period in men aged 20–30 years (9, 16, 17). Notably, intraprostatic DHT concentrations in BPH patients often exceed those in age-matched healthy individuals. This may stem from upregulated 17β-hydroxysteroid dehydrogenase (17β-HSD) activity, which converts androstenedione to DHT (18). Consequently, the exclusive attribution of BPH pathogenesis to androgens is inconsistent with clinical evidence. The mechanisms underlying androgen-independent BPH thus represent a current research priority.

Estrogen-related pathways partially resolve this paradox. In specific contexts, estrogens may exert more significant effects than androgens on BPH progression. As noted, aging reduces systemic androgen levels and alters the testosterone-to-estrogen ratio, elevating estrogenic activity. This shift is mechanistically linked to BPH pathogenesis (19).

Some studies suggest that alterations in the androgen-to-estrogen ratio are critical factors in BPH development (20). Yang et al. reported elevated serum levels of estrone (E1) and estradiol (E2) in BPH patients (21). The *CYP17A* gene may represent a potential genetic target for BPH intervention (22).

The estrogen hypothesis and DHT/5α-reductase pathway constitute two distinct mechanisms of prostate growth, exhibiting partial overlap. The estrogen pathway may offer a more effective therapeutic approach for BPH in populations with reduced steroid 5α-reductase 2 (SRD5A2) expression (23). Studies demonstrate that SRD5A2 deficiency induces significant changes in luminal epithelial cells (LE), while estrogen receptor 1 expression in LE cells increases inversely with SRD5A2 levels, suggesting an alternative mechanism for BPH pathogenesis (24). Estrogen-activated aquaporin 5 promotes epithelial-mesenchymal transition (EMT), thereby inducing BPH (25).

During prostate development, regulation may occur independently of AR but through estrogen-mediated pathways. In BPH progression, primary estrogen receptors include ERα, ERβ, and G protein-coupled estrogen receptor (GPER) (26).

Studies reveal that in normal prostate tissue, both ERα and ERβ are expressed in epithelial cells. In contrast, BPH tissue exhibits discontinuous ERα expression in epithelia, while ERβ shows increased expression clustered within hyperplastic nodules, indicating distinct pathogenic roles for these receptors (27).

ERα negatively correlates with androgen expression and regulates branching morphogenesis, stromal cell/extracellular matrix dynamics, and prostate stem cell activity, promoting progenitor cell proliferation. ERβ primarily mediates luminal epithelial cell differentiation and modulates progenitor cell proliferation (26). Agonists targeting ERβ (e.g., selective estrogen receptor modulators or ERβ-selective ligands) suppress castration-resistant BPH cell proliferation (28, 29) and inhibit AR signaling (30).

While ERα and ERβ represent classical research pathways in prostatic hyperplasia, the role of GPER is equally significant.GPER is highly expressed in prostate cells. Its activation inhibits proliferation in BPH-1, RWPE-1, and WPMY-1 cell lines. Mechanistically, GPER stimulation enhances yes-associated protein 1 (YAP1) phosphorylation and degradation, thereby suppressing prostatic hyperplasia (30).

Elevated 17β-estradiol (17β-E2) levels activate GPER via the Hippo-YAP1 pathway, exacerbating BPH and associated symptoms (31). Additionally, GPER/Gαi signaling interacts with EGFR/ERK and HIF-1α/TGF-β1 pathways to promote BPH progression (32). Although GPER’s precise mechanism in BPH remains unclear, its therapeutic potential is substantial, warranting further investigation.

### Insulin-related factors

BPH is increasingly recognized as a metabolic disorder linked to endocrine and metabolic dysregulation, with insulin-related pathways being central to its pathogenesis. Hyperinsulinemia heightens sympathetic nervous activity, increasing prostatic smooth muscle tone and exacerbating BPH symptoms. Both hyperinsulinemia and its associated obesity and metabolic syndrome are established risk factors for BPH (33).

Studies indicate that long-term use of anti-5α-reductase drugs (e.g., finasteride), while inhibiting DHT synthesis and alleviating BPH symptoms, may induce insulin resistance (IR) (34). Concurrently, IR can trigger hyperinsulinemia, potentiating a pathological cycle that worsens BPH. This mechanism may explain symptom progression in some BPH patients despite pharmacotherapy.

Hyperinsulinemia further stimulates IGF production. Elevated levels of IGF and IGF-binding protein (IGFBP) are risk factors for BPH, and IGF-I promotes prostate epithelial cell proliferation via specific target genes (35). Upon binding to IGF receptors, insulin induces prostate cell proliferation (36). Activation of the MEK/ERK pathway also enhances insulin-induced EMT, accelerating BPH progression (37). Therefore, IGF represents a promising therapeutic target for BPH, though further research is still needed to elucidate its underlying mechanisms.

### Thyroid-related hormones

Thyroid hormones (THs) regulate cellular growth, with emerging studies highlighting their association with BPH. Miro et al. demonstrated that THs modulate EMT and synergize with androgens to promote prostate cell proliferation. Notably, thyroid deiodinase enzymes may drive BPH progression toward prostate cancer (38).

Lee et al. further established that free thyroxine (FT4) levels significantly correlate with total prostate volume at specific testosterone thresholds (39). Heo et al. proposed thyroid-stimulating hormone (TSH) as a potential therapeutic target for BPH (40).

**Figure 1:**
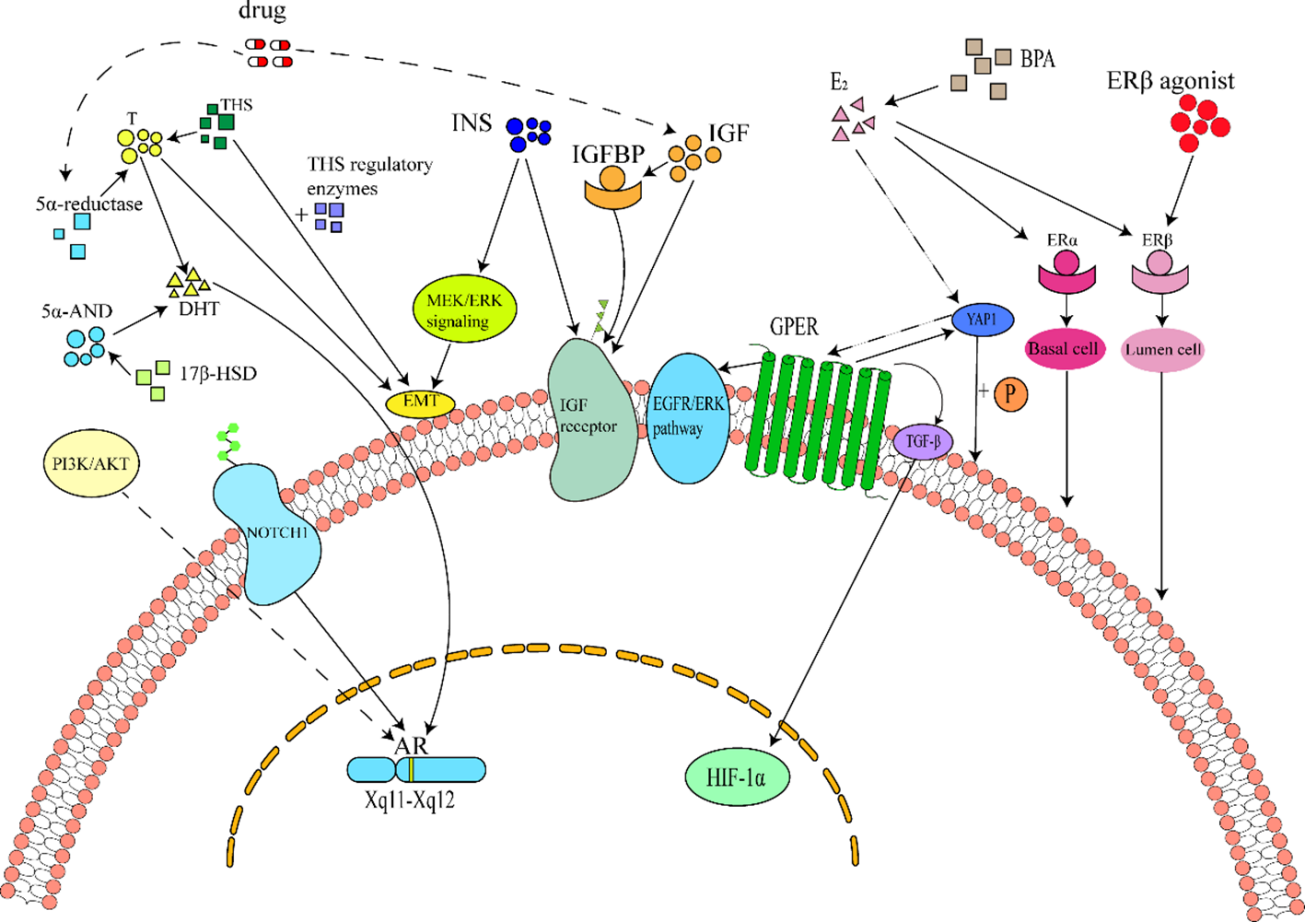
Schematic diagram of cellular and molecular mechanisms of hormones in BPH. ^1^Hormonal Pathways in Benign Prostatic Hyperplasia (BPH). Androgens, insulin, estrogens, and thyroid hormones constitute key regulators of BPH. Among these, androgens are the primary mediators of prostate pathogenesis, acting via androgen receptor (AR) activation by dihydrotestosterone (DHT). DHT is synthesized from testosterone through 5α-reductase catalysis or from 5α-androstanediol via 17β-hydroxysteroid dehydrogenase (17β-HSD) . AR signaling can also be activated independently of androgens through the Notch1 pathway and PI3K/AKT axis, further promoting prostate cell proliferation. Consequently, 5α-reductase inhibitors (e.g., finasteride) are widely used to suppress DHT production in BPH treatment. However, these drugs may induce adverse effects such as venous thromboembolism (VTE) or insulin resistance (IR) , leading to compensatory hyperinsulinemia and elevated insulin-like growth factor (IGF)/IGF-binding protein (IGFBP) levels. Insulin, IGF, and IGFBP collectively activate IGF receptors, driving BPH progression. Insulin additionally exacerbates BPH by inducing epithelial-mesenchymal transition (EMT) through MEK/ERK signaling. Estrogens, primarily 17β-estradiol (E2), exert dual effects via estrogen receptor subtypes: ERα, ERβ, and G protein-coupled estrogen receptor (GPER). ERα activation promotes stromal cell proliferation, whereas ERβ suppresses luminal epithelial cell growth . Elevated E2 activates GPER through the Hippo-YAP1 pathway and directly stimulates prostate proliferation via GPER/Gαi signaling, HIF-1α/TGF-β cascade, and EGFR/ERK axis. Thyroid hormones (THs) synergize with testosterone or modulate deiodinase enzymes to potentiate prostate cell hyperplasia.

Collectively, these studies indicate that thyroid-related hormones substantially influence BPH initiation and progression, though mechanistic clarity remains limited. Proposed pathways include PI3K/AKT axis activation and ERK1/2 signaling (39), providing a rationale for targeted BPH therapies. Thus, further research is imperative to elucidate these mechanisms and refine therapeutic strategies. The interplay of hormonal pathways in BPH pathogenesis is illustrated in **Figure 1**.

Additionally, deficiencies in luteinizing hormone (LH), follicle-stimulating hormone (FSH) (38), and prolactin (41) may constitute risk factors for BPH, warranting further investigation.

### Inflammation

While sex hormones critically regulate BPH, inflammation not only exacerbates hormone-dependent pathways but also emerges as a pivotal mechanism in hormone-independent BPH development (42).

Inflammation represents the body’s immune response to diverse stimuli (43), Chronic inflammation frequently accompanies histologically confirmed BPH. Clinical studies demonstrate that patients with inflammatory signatures exhibit a sevenfold higher risk of symptomatic progression compared to those without inflammation, with inflammatory severity correlating positively with prostate volume enlargement (44). During tissue repair processes, chronic inflammation induces microenvironmental alterations that activate pro-proliferative pathways. These include: (1) Epithelial barrier disruption, facilitating paracrine signaling; (2) DNA damage from OS and cytokine release; (3) Sustained cell proliferation mediated by growth factors (45).

Furthermore, inflammatory stimuli promote YAP1 activation via the RhoA/ROCK1 pathway, accelerating BPH progression and prostatic fibrosis (46, 47). Intriguingly, emerging evidence suggests that chronic inflammation—rather than androgen signaling—may initiate BPH pathogenesis. As proposed by Devlin et al., androgen-related factors are not primary etiological drivers, but inflammation triggers the disease cascade (48).

Inflammatory cytokines such as interleukin-1 (IL-1) have been found to promote BPH by affecting AR receptors (49). In addition to IL-1, many other related cytokines (50–54) also play important roles, including interleukin-8 (IL-8), tumor necrosis factor (TNF), receptor-associated factor 6 (TRAF6), interferon-γ (IFN-γ), interleukin-17(IL-17), transforming growth factor-β (TGF-β), interleukin-8(IL-8), Interferon-γ-inducible protein 10(CXCL10),interleukin-6(IL-6), interleukin-15(IL-15), and others.

The antagonism of TNF can reduce macrophage-mediated inflammation, TRAF6 can affect the proliferation of prostate cells through the Akt/mTOR signaling pathway, CXCL10 is overexpressed in the prostate, IL-8 induces autocrine or paracrine proliferation of BPH cells, IL-6 and IL-17 promote fibromuscular growth by inducing COX-2 expression, IL-17 can regulate the mPGES-1/PPAR-γ pathway in monocytes and macrophages, IL-15 can induce the proliferation of T lymphocytes, IFN-γ promotes the production of IL-15, and TGF-β promotes the mesenchymal-epithelial cell axis response, among others.

However, this perspective requires refinement, as chronic inflammation is not universally observed in BPH patients. Notably, anti-inflammatory therapies may exacerbate LUTS in some cases (55). Studies indicate that imbalances in steroid hormones (e.g., testosterone and estradiol) upregulate pro-inflammatory genes *Spp1* (encoding osteopontin) and *Saa1*. Elevated osteopontin exacerbates prostate cell proliferation (56), suggesting that hormone-related factors may occur before inflammation, and that the stimulatory effects of growth factors on prostate cells are initially observable (48), highlighting their dual roles in early BPH pathogenesis.

The interplay between sex hormones and inflammation is bidirectional: (1) Sex hormones modulate inflammation: Androgens suppress cytokine production, whereas estrogens influence immune cell recruitment. (2) Inflammation alters hormonal signaling: Inflammatory cytokines (e.g., TNF-α) inhibit AR activity and downregulate AR target genes (57). COX-2-derived PGE2 activates ADAM17, catalyzing testosterone shedding and activating ERK1/2 in BPH (58). Thus, inflammation synergizes with—rather than dominates—hormonal pathways in BPH etiology.

Additional inflammation-related mechanisms include: (1) Immunoregulatory receptors: Sialic acid-binding Ig-like lectin-1 (Siglec-1) modulates leukocyte adhesion (59). (2) Natural compound targets: Tanshinone IIA (Tan IIA) and baicalin regulate ERK1/2 signaling (60). (3) Key signaling pathways: NF-κB/MAPK, JAK-STAT, PI3K-Akt, and AMPK cascades (61). (4) Tight junction disruption: Downregulation of Claudin-1 compromises epithelial barrier function (62).

Conclusion: Inflammation independently influences BPH through cytokines and interacts with hormonal axes, growth factors, and signaling pathways. While anti-inflammatory strategies hold promise, their clinical application requires caution due to potential adverse effects on LUTS. Future research should prioritize dual-target therapies that concurrently modulate hormonal and inflammatory drivers. Key inflammatory mechanisms and their interactions are detailed in **Table 2**.

**Table 2:** Mechanisms associated with BPH inflammation.

Inflammation related factors	Non-inflammatory factor-related	Inflammatory factor related
	RhoA/ROCK1 pathway → YAP1 activation → BPH deterioration	IL-1→AR→BPH
	ITLN-1	TNF→macrophages→BPH inflammation worsening
	TanIA and Ba regulate ERK1/2 signaling	TNEF-α→repression of AR and AR target genes
	NF-kB/MAPK/JAK-STAT/PI3K-Akt and AMPK pathways	TRAF6 → Akt/Mtor → prostate cell proliferation
	Down-regulation of the connexin Claudin-1	CXCL10 overexpression → autocrine or paracrine proliferation of BPH cells
	Apoptosis	IL-6 →COX-2 → Fibromuscular growth/PGE2-ADAM-17 → testosterone level effects/TGF-a shedding → ERK1/2 pathway activation
	,growth factors	IL-17→COX-2 →Fibromuscular growth/Mpges-1/PPAR-1 pathway
	cellular fibrosis	IL-15→Growth of T-lymphocytes
		IFN-γ→ prompts L-15 generation
		TGF-β→ mesenchymal-epithelial cell axis response

### Other relevant mechanisms and factors

Microbiota dysbiosis significantly contributes to BPH pathogenesis through intertwined inflammatory and hormonal axes. Specific bacterial populations—including *Lactobacillus*, acetylated bacteria (*Acinetobacter*), *Flavobacterium*, *Oscillibacter*, *Pseudoflavonifractor*, *Enterococcus*, and butyrate-producing bacteria—exhibit close correlations with BPH progression (63).

Prostate-specific flora dysbiosis (e.g., *Pseudomonas* dominance) activates NF-κB signaling, inducing EMT and suppressing apoptosis. This cascade promotes prostatic cell proliferation and stromal remodeling (64). Concurrently, gut microbiota dysbiosis exacerbates intraprostatic inflammation, with *Escherichia coli* being a primary driver. *E. coli* lipopolysaccharides activate NF-κB in prostate epithelial cells, amplifying cytokine release and leukocyte infiltration (65).

Metabolomic imbalances further potentiate BPH pathogenesis: (1) Short-chain fatty acid (SCFA) depletion compromises intestinal barrier integrity, enabling microbial-associated molecular patterns (MAMPs) to trigger systemic inflammation (66); (2) Hormonal axis disruption occurs via GPER dysregulation, linking microbiota-derived signals to estrogenic pathways in prostatic stroma (67).

Beyond gut microbiota, the urinary tract microbiota significantly contributes to BPH pathogenesis. Specific urethral microbes—including *Ureaplasma urealyticum*, *Haemophilus*, *Staphylococcus*, *Granulicatella*, and *Listeria*—demonstrate strong correlations with BPH severity (68). Although microbiota-related mechanisms interact with inflammatory and hormonal pathways, interventions targeting the intestinal microenvironment alone can ameliorate BPH progression, underscoring its independent role in disease modulation (69).

Apoptosis, growth factors and cellular fibrosis are closely related to BPH. The equilibrium between prostate tissue proliferation and apoptosis impacts BPH development. Once this balance is disrupted, it can trigger a series of pathological changes in prostate cells (70). When apoptosis is resistant, BPH may even transform into prostate cancer (71). Anti - apoptotic protein Bcl - 2 and pro - apoptotic protein BAXS are two proteins directly linked to BPH. CASP3 is also crucial for regulating mitochondrial - mediated apoptosis. Bcl - 2 and BAX, as upstream regulators of CASP3, can affect its activity and also serve as CASP3 substrates to impact downstream processes (72). Studies on canine models have found that in castrated prostates, the only observed cell death was apoptosis and tumorous forms (73).

Androgens, cytokines, and growth factors are all associated with an imbalanced cell proliferation/apoptosis ratio in prostate cells. As previously mentioned, hormone - related growth factors like IGF and EGF can promote BPH development through hormone - related signaling pathways (32, 35). In addition to these factors, basic fibroblast growth factor (bFGF), vascular endothelial growth factor (VEGF), nerve growth factor (NGF), and platelet - derived growth factor (PDGF) also help maintain and promote prostate cell proliferation (74). The inflammatory process is thought to be an initial link between growth - factor - induced proliferation and glandular remodeling in BPH (48).

Cellular fibrosis is a key pathological mechanism in BPH clinical progression and can cause mainstream drug treatment failure. It is influenced by genetic factors, microbial invasion, inflammation, lifestyle, external stimuli, and aging. Microorganisms contribute to BPH progression by chronic inflammation, which promotes prostate fibrosis and hyperplasia through ECM deposition. This is associated with inflammation - induced EMT, myofibroblast differentiation, ECM accumulation, and increased tissue stiffness (75). Cellular fibrosis is still influenced by hormone - related signaling pathways. Estrogen - related signaling pathways can increase the proliferation of prostate stromal cells and stromal fibrosis (32).

Notably, these mechanisms remain less extensively studied than hormonal and inflammatory pathways. Emerging evidence implicates OS (e.g., Prdx3 dysregulation), autophagy impairment, and pyroptosis in BPH pathogenesis, though their interactions with canonical drivers (e.g., androgen signaling, NF-κB activation) require further elucidation. A comprehensive understanding of BPH must integrate these multifactorial interactions to develop targeted therapeutic strategies.

## Autophagy’s mechanism of action in BPH

Autophagy plays a crucial role in maintaining cellular homeostasis and contributes to cell death pathways. Depending on the mechanisms mediating cargo delivery to lysosomes, autophagy is classified into three main types: macroautophagy, chaperone-mediated autophagy (CMA), and microautophagy. The regulatory mechanisms governing autophagy are complex, involving upstream signaling pathways primarily consisting of mTOR-dependent and mTOR-independent axes (76).

Macroautophagy (frequently termed simply as autophagy) involves the coordinated action of autophagy-related (ATG) proteins to sequester cargo within double-membrane vesicles termed autophagosomes (77). Subsequent fusion of autophagosomes with lysosomes forms autolysosomes, where cargo is degraded by lysosomal hydrolases. In contrast, both CMA and microautophagy occur independently of core ATG proteins for their initiation and execution. Cargos targeted by autophagy are diverse and include damaged organelles such as the endoplasmic reticulum (ER) and mitochondria, as well as cytoplasmic components, protein aggregates, and invading pathogens (78). Originating from various sources like the ER, plasma membrane, recycling endosomes, mitochondria, ATG9 vesicles, COPII vesicles, and ER-Golgi intermediate compartment, isolation membranes form autophagosomes. Autophagy also mediates selective clearance of specific organelles, with mitophagy (mitochondria) and ER-phagy being notable examples in the context of BPH (79).

In CMA, individual cytosolic proteins carrying a KFERQ-like motif are recognized by the cytosolic chaperone HSPA8/HSC70 and delivered to the lysosomal membrane. There, they bind specifically to monomeric lysosome-associated membrane protein type 2A (LAMP2A), triggering LAMP2A multimerization to form a translocation complex. The substrate protein is then unfolded and gains access to the lysosomal lumen for degradation (80).

Microautophagy involves the direct uptake of cytoplasmic material through invagination or involution of the lysosomal membrane itself, categorized into ESCRT-dependent and ESCRT-independent modes, which are beyond the scope of this discussion (80).

Autophagic flux, measuring the complete degradation process, is a critical indicator of autophagic activity. Observations indicate a reduction in autophagic flux in BPH patients (81). Studies demonstrate that inhibiting the LLGL2 gene enhances autophagy, subsequently suppressing prostate cell proliferation (82).Moreover, knockdown of FOXK1 promoted autophagy and attenuated TGF-β-induced EMT and epithelial cell proliferation (83).

Paradoxically, other studies suggest that inhibiting autophagy can promote prostate cell apoptosis. For instance, inhibiting Beclin-1 suppressed autophagy and initiated cell apoptosis, effectively inhibiting prostate cell proliferation (84). Some studies also report that autophagy supports the growth of prostate stromal fibroblasts (85).

Collectively, these findings indicate that autophagy exerts divergent effects on BPH progression depending on the specific cellular context and experimental conditions.

The prevailing interpretation is that autophagy primarily serves a protective function under stress, degrading cellular components to recycle biomolecules, potentially reducing metabolic burden and creating space for cell survival—an effect potentially advantageous in the hypercellular environment of BPH.

Supporting this, one study observed prostate cells undergoing androgen deprivation (AD). In the early stages of AD, autophagy exhibited a compensatory cytoprotective function. Throughout the process, autophagy antagonized apoptosis, playing a survival role. Critically, inhibiting autophagy suppressed cell growth under these conditions (86).

However, the selective degradation of essential organelles through mechanisms like mitophagy and ER-phagy carries risks. Excessive removal of mitochondria, vital for energy production, could compromise cellular metabolism and potentially accelerate apoptosis pathways.The precise regulatory mechanisms determining when selective autophagy is protective versus detrimental in BPH remain incompletely elucidated. Elucidating these mechanisms may resolve apparent contradictions regarding autophagy’s effects in similar or different contexts.

Numerous factors concurrently influence biological processes *in vivo*. In other proliferative disorders, autophagy similarly exhibits context-dependent effects, acting either as a promoter or suppressor, and is not unique to BPH. Consequently, research focuses on harnessing autophagy pathways for therapeutic benefit against various tumors (87). Regarding BPH pathogenesis, studies report downregulation of autophagy in patients correlates with increased expression of ERα (88).

Rodent experiments using 5α-reductase inhibitors revealed that enhanced autophagy was accompanied by suppression of AR signaling (89).Furthermore, autophagy can counteract inflammatory mediators and suppress detrimental inflammatory pathways (89).

**Figure 2:**
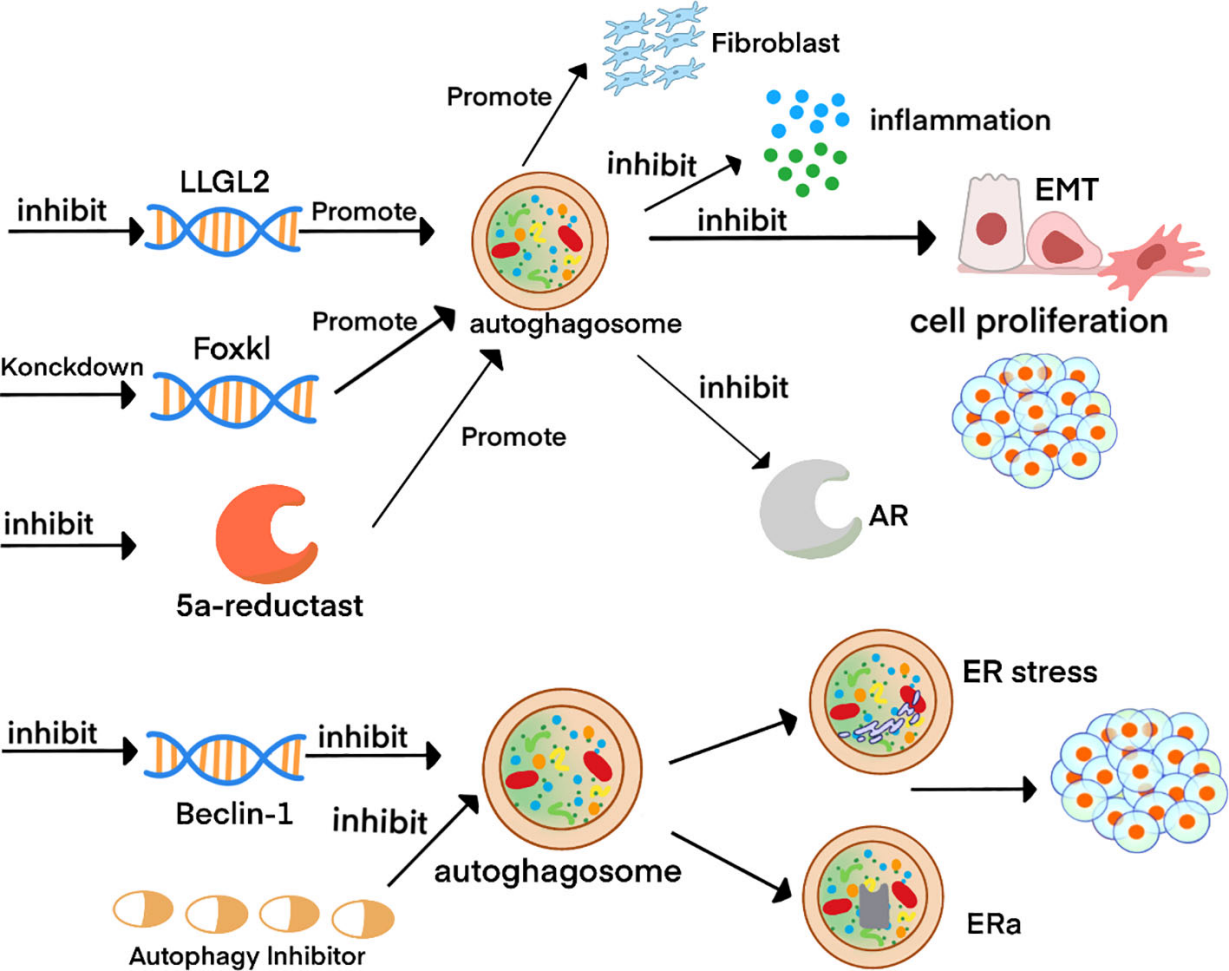
Schematic diagram of autophagy-related molecular mechanisms in BPH. ^2^Inhibition of the *LLGL2* gene combined with FOXK1 knockdown enhances autophagy in prostate tissue. This augmentation attenuates the inflammatory response, suppresses epithelial-mesenchymal transition (EMT), and reduces epithelial cell proliferation, while paradoxically promoting the growth of prostate fibroblasts. Conversely, suppression of the *Beclin-1* gene or pharmacological inhibition of autophagy induces endoplasmic reticulum (ER) stress, elevates ERα expression, and ultimately drives cell proliferation.

As previously discussed, ERα, AR, and inflammatory factors are all established risk factors in BPH, and autophagy demonstrably suppresses both pathways. Collectively, these findings support the contention that maintaining a baseline level of autophagy contributes positively to BPH stability. Nevertheless, the underlying molecular mechanisms remain largely unexplored, and *in vivo* evidence is still scarce—representing critical areas for future investigation and methodological refinement.The dual roles of autophagy in BPH progression are schematically depicted in **Figure 2**.

## The mechanism of action of OS on BPH

BPH is an age-progressive disease, and OS significantly contributes to age-related pathologies (90). This suggests a potential mechanistic link between BPH and OS. Notably, OS levels are markedly elevated in BPH patients compared to age-matched healthy individuals. Epidemiological studies indicate that heightened OS—even when secondary to cardiovascular disease—correlates with increased risk of prostate pathologies (91).

OS pathogenesis involves excessive production of reactive oxygen species (ROS) and reactive nitrogen species (RNS), dysregulated antioxidant enzyme activity, lipid peroxidation, and protein carbonylation. Accumulated ROS disrupt the cellular antioxidant defense system, depleting key enzymes, oxidizing proteins, and reducing critical cytokines. This shifts tissues from redox balance to an oxidized state, inducing cellular and tissue damage. Concurrent RNS accumulation activates apoptosis via multiple pathways, including NF-κB, MAPK, Nrf-2/Keap-1/ARE, and PI3K/Akt (92).OS exerts dual pathological mechanisms (93): (1) Direct macromolecular damage by reactive radicals (e.g., OH·, ONOO^-^, HOCl), causing cellular dysfunction or death through oxidation of lipids, proteins, and DNA; (2) Dysregulated redox signaling mediated by molecules like H_2_O_2_, which disrupt kinase/phosphatase activity and gene expression.

Both mechanisms can coexist in disease contexts like BPH.

Theoretically, OS could suppress proliferation by inducing cell death. In BPH, this might promote apoptosis over proliferation, suggesting a protective role. Paradoxically, OS also acts as a key risk factor through alternative mechanisms: (1) OS directly stimulates prostate cell proliferation by activating MAP kinases and PI3K/AKT signaling (94). (2) Experimental models demonstrate that OS-induced prostate damage increases prostate weight, epithelial hyperplasia, stromal thickening, and fibrosis (95).

Supporting the therapeutic targeting of OS: Studies have demonstrated that activation of the Nrf2/ARE pathway alleviates prostatic hyperplasia in BPH rat models by suppressing OS (96).

Nuclear factor erythroid 2-related factor 2 (Nrf2) further modulates BPH progression by enhancing antioxidant responses, promoting apoptosis, and inhibiting macrophage-mediated inflammation (96). Additionally, Wang et al. revealed that BPH-induced testicular damage involves OS-driven m6A modification dysregulation and autophagic flux impairment (97). Collectively, these studies underscore OS inhibition as a promising therapeutic strategy for BPH.

However, the molecular mechanisms underlying the correlation between OS and BPH remain incompletely elucidated. Beyond the aforementioned pathways, other molecular mechanisms contribute to OS-BPH interactions: Enhanced expression of prostate-associated gene 4 (PAGE4) under OS conditions activates phosphorylated ERK1/2 (p-ERK1/2) while suppressing phosphorylated JNK1/2 (p-JNK1/2), thereby inhibiting apoptosis (98); the IGF-1/PI3K/AKT/FOXO axis attenuates prostate cell proliferation by modulating OS responses and apoptotic signaling (99); and xanthine oxidase (XO)/JAK/STAT signaling promotes OS pathogenesis via ROS generation (100).

In BPH pathogenesis, inflammation induces cellular and genomic damage yet paradoxically triggers compensatory proliferation and enhanced replication. This dual role arises from bidirectional crosstalk between inflammation and OS: Inflammatory cells (e.g., macrophages, T-cells) release excessive ROS, driving OS accumulation (101); conversely, OS-derived metabolic byproducts (e.g., lipid peroxides) activate pro-inflammatory factors including NF-κB, AP-1, and TNF-α, perpetuating chronic inflammation (102). Consequently, OS is a pivotal driver of inflammatory BPH progression.

Hormonal pathways further integrate with OS signaling: OS disrupts thyroid hormone synthesis and testosterone metabolism, while estrogen counteracts OS through antioxidant activity (103). Pharmacological inhibition of NADPH oxidase (NOX) by apocynin (Apo) reduces OS and concurrently suppresses AR signaling, ameliorating BPH via the AR/TGF-β/NOX4 axis (104). Notably, in transgenic mice with prolactin-mediated androgen signaling deficiency, antioxidant therapy remained effective, highlighting OS inhibition as a viable strategy for androgen-independent BPH (105).

Several OS-targeting drugs demonstrate therapeutic efficacy in BPH: Berberine (BBR; reduces serum testosterone/DHT and inhibits OS) (106); palmitoylethanolamide (um-PEA) and baicalein (Baic; modulate AR signaling and attenuate OS) (107).

**Figure 3:**
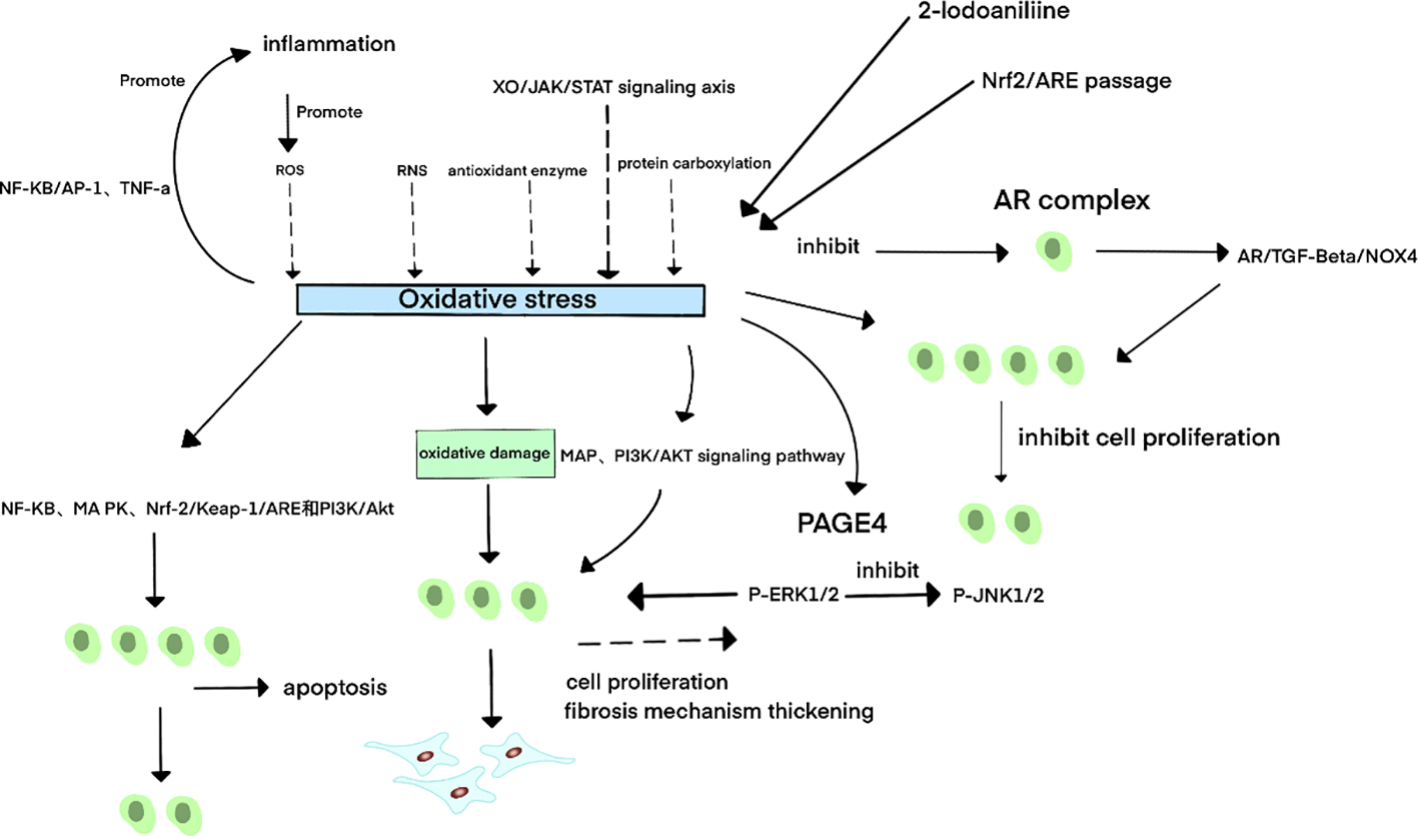
Schematic diagram of oxidative stress-related mechanisms in BPH. ^3^Oxidative stress (OS) primarily arises from excessive production of reactive oxygen species (ROS) and reactive nitrogen species (RNS), dysregulated antioxidant enzyme activity, lipid peroxidation, and protein carbonylation. The xanthine oxidase (XO)/JAK/STAT signaling axis further contributes to OS pathogenesis. A bidirectional relationship exists between OS and inflammation: Inflammatory cells release excessive ROS, driving OS accumulation; conversely, OS-derived metabolites activate pro-inflammatory factors such as NF-κB, AP-1, and TNF-α, perpetuating chronic inflammation. In benign prostatic hyperplasia (BPH), OS exhibits dual regulatory effects:Pro-apoptotic mechanisms: OS induces apoptosis through NF-κB, MAPK, Nrf-2/Keap-1/ARE, and PI3K/Akt signaling pathways, thereby suppressing prostate cell proliferation. Pro-proliferative mechanisms: OS directly stimulates prostate cell growth by activating MAPK and PI3K/AKT signaling. Under OS conditions, upregulation of prostate-associated gene 4 (PAGE4) activates phosphorylated ERK1/2 (p-ERK1/2) while inhibiting phosphorylated JNK1/2 (p-JNK1/2), thereby reducing apoptotic cell death and promoting cellular survival.

Despite these benefits, OS exerts context-dependent effects on BPH—potentially suppressing proliferation through cell death induction while exacerbating disease via inflammation and hormonal dysregulation. The dual regulatory effects of OS and its crosstalk with inflammation are shown in **Figure 3**.

However, Savas et al. reported no direct correlation between plasma antioxidant status and BPH in untreated patients (108). This discrepancy underscores the need for multifactorial analyses to delineate OS’s contextual impacts and guide therapeutic development.

Multiple strategies exist to inhibit OS, most of which suppress prostate cell proliferation. For example, administration of diphenyliodonium (DPI) or modulation of the Nrf2/ARE pathway exerts antiproliferative effects by scavenging ROS and enhancing antioxidant responses; Nrf2 activation additionally suppresses macrophage-mediated inflammation. Furthermore, pharmacological inhibition of OS via the NOX inhibitor apocynin (Apo) downregulates AR expression, thereby ameliorating BPH through the AR/TGF-β/NOX4 signaling axis.

## The role of pyroptosis in prostate hyperplasia

Pyroptosis is a gasdermin-mediated programmed cell death characterized by lytic rupture and inflammatory activation. Under physiological conditions, it functions as a host defense mechanism against pathogens; conversely, under pathological conditions, it drives chronic inflammation, tissue damage, and cytokine storms (109).

This lytic cell death is defined by gasdermin-dependent pore formation in the plasma membrane, which facilitates the release of pro-inflammatory cytokines (e.g., IL-1β, IL-18, IL-33) and intracellular components such as lactate dehydrogenase (LDH), ultimately leading to cell lysis (110).

Pyroptosis is primarily induced through four distinct pathways: (1) Canonical inflammasome pathway; (2) Non-canonical inflammasome pathway; (3) Apoptotic caspase-mediated pathway;(4) Granzyme-triggered pathway (111).

Key molecular executors include: (1) Inflammatory caspases (caspase-1, -4, -5, -11) activated by inflammasomes; (2) Apoptotic caspases (caspase-3, -6, -8) that cleave gasdermins under specific stimuli; (3) Granzymes (granzyme A/GZMA, granzyme B/GZMB) secreted by cytotoxic lymphocytes (112).

Both canonical and non-canonical pathways converge on gasdermin D (GSDMD) cleavage by inflammasomes, initiating pyroptosis. Major inflammasomes include NLRP3, NLRC4, AIM2, and Pyrin, which are cytoplasmic pattern recognition receptors activated by pathogen-associated molecular patterns (PAMPs) or damage-associated molecular patterns (DAMPs) (113). Caspases exist as inactive zymogens and require specific triggers for activation: inflammatory caspases respond to infection or danger signals (114), while apoptotic caspases are engaged in cell death programs.

Inflammasomes are cytoplasmic pattern recognition receptors (PRRs). Their activation is associated with PAMPs and DAMPs. Once inflammasomes recognize these molecular patterns, they become activated and trigger an inflammatory response. The recognition of PAMPs can activate inflammasomes through both canonical and non - canonical pathways (115).

In the canonical pathway, inflammasomes are activated by NOD - like receptors (NLRs) or PYHIN proteins.In the canonical pathway, inflammasome formation is initiated by NLRs or PYHIN proteins. This activation leads to caspase-1 activation, which promotes the maturation of IL-1β and IL-18 (116). GSDMD, cleaved by caspase-1, generates an N-terminal fragment (GSDMD-NT, ~30 kDa). Upon oligomerization, GSDMD-NT inserts into the plasma membrane to form pores, resulting in pyroptotic cell rupture and the release of inflammatory cytokines and other cellular molecules (117).

In the non-canonical pathway, caspase-4/5 in humans (and its functional homolog caspase-11 in mice) catalyze the maturation of pro-IL-18 to IL-18 (118). Murine caspase-11 and caspase-5 (which shares structural similarity with human caspase-5) are also implicated in the secretion and release of IL-1β (119). Importantly, the maturation and release of both IL-1β and IL-18 via the non-canonical pathway still depend on the NLRP3 inflammasome and caspase-1. Their activation is triggered by K^+^ efflux through GSDMD-NT pores formed during pyroptosis (120).

Caspase-4 is a functional homolog of caspase-11, while caspase-11 and caspase-5 exhibit structural similarity. Upon infection with Gram-negative bacteria, intracellular LPS levels increase. Both caspase-11 and caspase-4 can be directly activated by LPS. In contrast, caspase-5 is activated by LPS and IFN-γ (121, 122).

Apoptotic caspases indirectly induce pyroptosis: (1) Caspase-3 cleaves GSDMD or GSDME during chemotherapy, but mannitol metabolite GlcNAc-6P blocks this process via AMPK-mediated GSDME phosphorylation (123); (2) Caspase-8 activation by Yersinia effector YopJ triggers NLRP3-dependent pyroptosis (124); (3) Caspase-6 potentiates NLRP3/caspase-1-mediated pyroptosis (125).

Granzyme-mediated pyroptosis predominantly involves GZMB, which activates caspase-3 to cleave GSDME, inducing pore formation (126). This mechanism underlies chimeric antigen receptor T cell (CAR-T) cytotoxicity (127).

Although pyroptosis research in BPH remains limited, accumulating evidence implicates its role: (1) Elevated IL-1β and IL-18 levels in BPH tissues (128); (2) High caspase-1 expression (71.4%) in BPH patients (129); (3) NLRP3 inflammasome activation in proliferating prostate cells (130). Proposed mechanisms include the P2X7R-NEK7-NLRP3 axis driving GSDMD-NT-mediated prostate epithelial pyroptosis (131) and enhanced caspase-3 activity in BPH tissues (132). Linghe Zang et al. (133) found that Qianliexin capsule can inhibit the activation of related inflammasomes.

**Figure 4:**
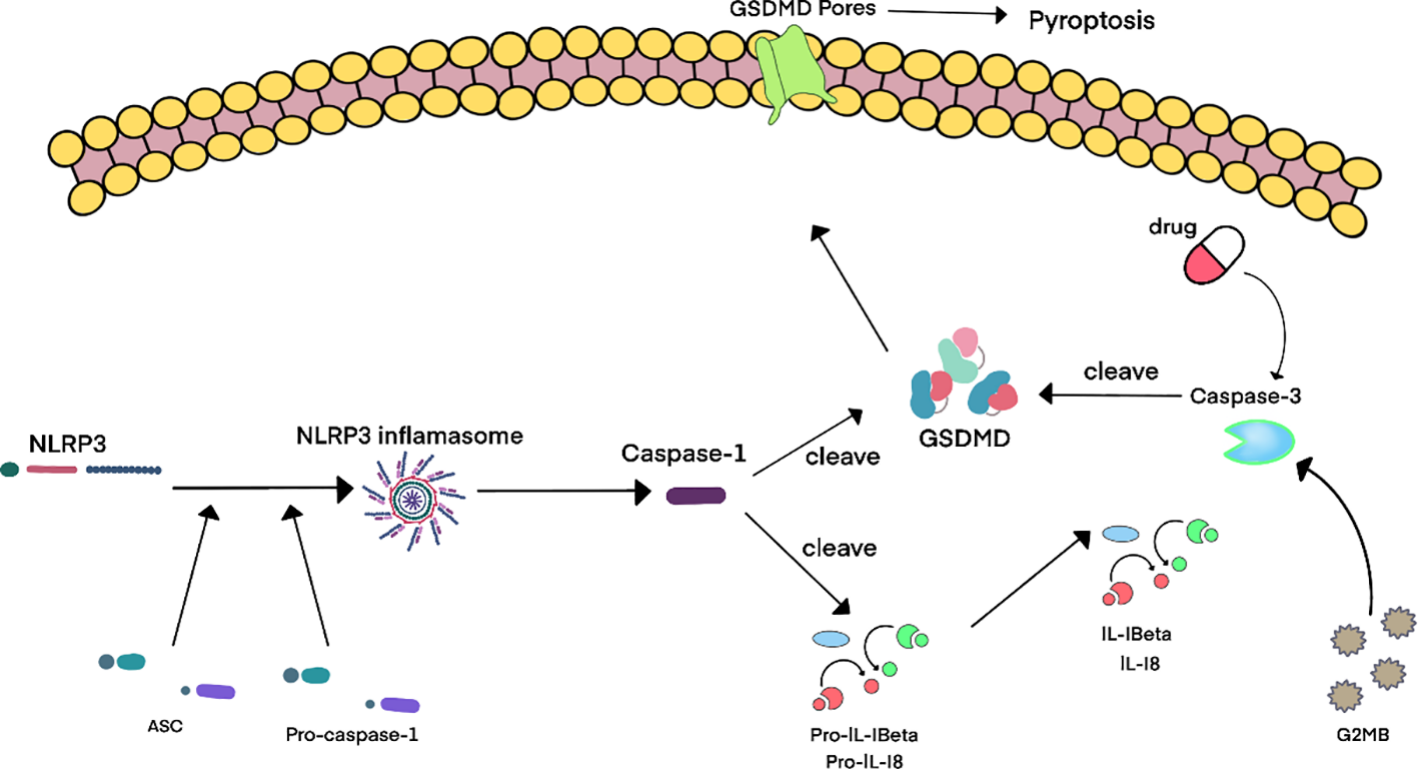
Schematic diagram of cellular and molecular mechanisms of pyroptosis in BPH. ^4^Accumulating evidence reveals upregulated expression of pyroptosis-associated molecules – including IL-1β, IL-18, NLRP3 inflammasome components, caspase-1, and caspase-3 – in benign prostatic hyperplasia (BPH) tissues. Current consensus posits that the canonical pyroptosis pathway is predominant in BPH, involving NLRP3 inflammasome activation which then triggers caspase-1-mediated cleavage of gasdermin D (GSDMD). This process induces pore formation and the release of inflammatory cytokines. Notably, pharmacological agents targeting GSDMD can lead to caspase-3 activation, thereby initiating caspase-3-mediated pyroptosis through cleavage of GSDME. Furthermore, granzyme B (GZMB), secreted by cytotoxic lymphocytes, activates caspase-3 to generate the N-terminal fragment of GSDME (GSDME-NT), which ultimately drives pyroptosis and amplifies the inflammatory cascade in BPH pathogenesis.

In summary, pyroptosis contributes to BPH pathogenesis through inflammasome-mediated cell death and chronic inflammation, warranting further mechanistic exploration. Molecular pathways of pyroptosis activation in BPH are summarized in **Figure 4**.

## The role of Prdx3 in prostate hyperplasia

Peroxiredoxins (PRDXs) are a family of antioxidant enzymes that catalyze hydrogen peroxide (H_2_O_2_) reduction to suppress OS. Six mammalian isoforms exist (PRDX1-PRDX6), among which Prdx3 is uniquely localized to mitochondria and exhibits high abundance in human tissues (134). Mitochondria represent the primary source of ROS, including superoxide anion (O_2_^-^), H_2_O_2_, and hydroxyl radical (•OH). Prdx3 protects against ROS-induced cellular damage by scavenging mitochondrial H_2_O_2_ via its peroxidase activity (135).

Early studies demonstrated elevated Prdx3 expression in prostate cancer, where low Prdx3 levels correlate with significantly improved patient survival. Functionally, Prdx3 maintains mitochondrial integrity to protect against hypoxic damage (136) and directly regulates prostate cell proliferation *in vitro* (137). Prdx3 overexpression is further associated with aggressive phenotypes in breast, colorectal, gastric, and lung cancers (138).

Although benign tumors generally pose lower risks, BPH — despite its benign classification — imposes significant clinical burdens due to high prevalence and anatomical sensitivity. Consequently, investigating Prdx3 in BPH pathogenesis is warranted. Existing evidence confirms marked Prdx3 upregulation in BPH prostate tissues (139), supporting its role as a pro-proliferative driver in benign hyperplastic disorders.

Critically, Prdx3 intersects with OS, pyroptosis, and autophagy pathways in BPH: (1) Prdx3 suppresses autophagy by reducing mitochondrial ROS, thereby preventing autophagic clearance of damaged organelles; (2) Autophagy deficiency activates NLRP3 inflammasomes and caspase-1, inducing pyroptosis; (3) Pyroptotic cell death releases inflammatory cytokines (e.g., IL-1β, IL-18) that stimulate compensatory prostate cell proliferation.

Thus, Prdx3 represents a pivotal molecular nexus in BPH pathogenesis, integrating OS defense with inflammatory cell death and proliferative signaling.

## The impact of the interaction between Prdx3, autophagy, OS, and cell pyroptosis on prostate hyperplasia

**Figure 5:**
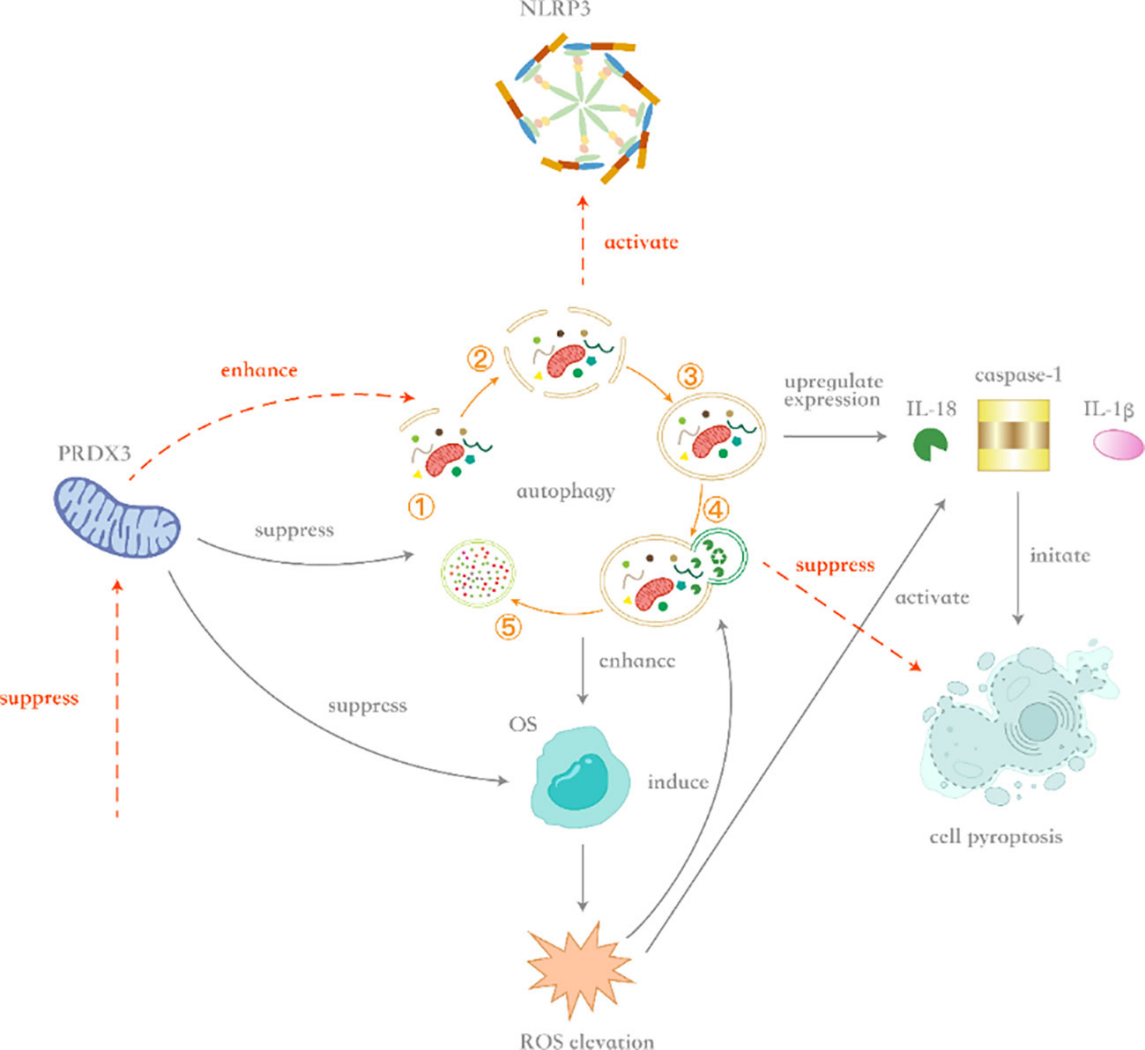
Schematic diagram of crosstalk mechanisms between Prdx3, oxidative stress, autophagy, and pyroptosis. ^5^Upon *Prdx3* upregulation, autophagy inhibition occurs alongside increased expression of interleukin-18 (IL-18), caspase-1, and interleukin-1β (IL-1β), thereby inducing caspase-1-dependent pyroptosis. Although *Prdx3* suppresses oxidative stress (OS) under physiological conditions, its inhibition of autophagy indirectly enhances OS accumulation in benign prostatic hyperplasia (BPH) . When OS intensity exceeds a threshold, reactive oxygen species (ROS) generation simultaneously triggers compensatory autophagy and activates the NLRP3 inflammasome. Enhanced autophagy counteracts OS through clearance of damaged organelles (e.g., via mitophagy), whereas sustained ROS elevation promotes caspase-1 activation via NLRP3, initiating pyroptosis. Conversely, *Prdx3* downregulation enhances autophagic flux and suppresses NLRP3 inflammasome assembly. In this context, augmented autophagy mitigates OS, while reduced NLRP3 activation attenuates pyroptosis, ultimately suppressing inflammatory-driven prostate hyperplasia.

The concluding hypothesis posits that Prdx3 inhibits autophagy, leading to relative enhancement of OS, which subsequently induces pyroptosis and promotes BPH pathogenesis. **Figure 5** illustrates the key mechanisms of Prdx3 in BPH: Prdx3 indirectly amplifies OS by suppressing autophagy, triggering pyroptosis and driving prostate cell proliferation. However, under certain conditions, Prdx3’s antioxidant capacity may be overwhelmed by OS from other mechanisms.

The interplay between autophagy and OS operates bidirectionally: (1) OS induces autophagy: Excessive ROS activate upstream autophagy regulators (AMPK, mTOR, MAPK, PI3K) and modify autophagy-related proteins, triggering unregulated autophagic flux that may culminate in cell death (140, 141); (2) Autophagy suppresses OS: By eliminating redundant organelles (particularly via mitophagy) and reducing mitochondrial DNA-mediated ROS production, autophagy maintains redox homeostasis. Defective autophagy exacerbates OS and inflammasome activation (142, 143).

In BPH, both processes exhibit “double-edged sword” effects on cellular proliferation: OS promotes apoptosis yet concurrently stimulates inflammatory cascades, while autophagy attenuates OS but may be incomplete in hyperplastic tissues. Theoretically, maintaining balanced autophagic flux could mitigate BPH progression.

Despite OS being objectively elevated in BPH tissues, prostatic cell proliferation predominates, indicating that compensatory mechanisms override OS-mediated growth suppression. Although exacerbating OS could theoretically inhibit proliferation, this approach is clinically impractical due to unpredictable systemic damage. Critically, incomplete autophagy in BPH perpetuates OS and pyroptotic signaling. Thus, sustaining competent autophagy is essential to stabilize OS and prevent pathological hyperplasia.

Prdx3 critically underpins autophagy-OS dysregulation in BPH: (1) Prdx3 upregulation inhibits mitophagy and suppresses ROS scavenging, paradoxically elevating mitochondrial OS despite its antioxidant role (144); (2) Prdx3 knockdown increases mitochondrial ROS but activates protective mitophagy and inhibits NLRP3 inflammasomes, reducing pyroptosis (144, 145); (3) In prostatic neoplasia, Prdx3 promotes cell survival by reducing OS, highlighting its context-dependent functions (146).

Mechanistically, Prdx3-driven autophagy impairment enhances OS, activating caspase-1 via NLRP3 inflammasomes and upregulating IL-1β/IL-18, thereby inducing pyroptosis (147). Concurrently, pyroptosis-released cytokines stimulate compensatory prostate cell proliferation (148). Therefore, in this process, Prdx3, autophagy, OS, and cell pyroptosis act synergistically in the pathogenesis of BPH. The upregulation of Prdx3 inhibits autophagy, leading to a relative enhancement of OS and the occurrence of cell pyroptosis, ultimately promoting the proliferation of prostate cells.The crosstalk between Prdx3, autophagy, OS, and pyroptosis is illustrated in **Figure 5**.

## Conclusion

The hallmark of BPH is the acceleration of cell proliferation over cell death. Under normal physiological conditions, the growth of organs and tissues depends on the dynamic balance between cell death and cell proliferation. When the intraprostatic microenvironment changes, disrupting this balance can lead to BPH. Such microenvironmental changes involve imbalances in inflammation, hormones, and other factors. To further explore the pathogenesis of BPH, it is crucial to understand the factors driving inflammation and hormonal alterations. Prdx3, OS, cell pyroptosis, and autophagy can all directly or indirectly influence the levels of hormones or inflammation.

However, Prdx3, OS, cell pyroptosis, and autophagy exhibit bidirectional regulatory effects in BPH, resulting in paradoxical outcomes. Regarding OS, numerous studies propose controlling BPH by inhibiting OS (149). This approach is based on the rationale that OS inhibition can reduce inflammatory factors, thereby suppressing prostatic hyperplasia. Consequently, enhancing antioxidant capacity and reducing inflammation appear to be promising strategies for managing this disease.

Theoretically, high levels of OS are detrimental in BPH, and administration of antioxidants should alleviate the disease. However, randomized clinical trials have frequently shown no significant benefit (150). Does this discrepancy arise from a flaw in the theoretical premise, or is it that antioxidant administration or enhancement of antioxidant capacity does not necessarily reduce OS?

Returning to the pathogenesis of BPH, prostatic tissues commonly exhibit elevated ROS levels and abundant inflammatory factors. Paradoxically, compensatory upregulation of antioxidants, such as Prdx3, is also frequently observed. The presence of substantial amounts of antioxidants indicates a robust compensatory antioxidant capacity in the prostate. Yet, OS signs persist, representing a key paradox.

OS induces autophagy. Autophagy, in response to stress, reduces and degrades organelles, a degradation process that promotes cellular adaptation to an inflammatory environment and enhances cell survival. This pro-survival effect represents a risk factor for BPH progression. However, in certain contexts, autophagy can also promote apoptosis. Prdx3 inhibits autophagy, and defective autophagy can lead to increased OS and inflammation. Paradoxically, although Prdx3 functions as an antioxidant to counteract excessive ROS, its inhibition of autophagy ultimately leads to a net increase in OS levels, which is followed by cell pyroptosis that results in enhanced inflammation.

Following BPH onset, the rate of cell death is lower than the rate of cell proliferation. Within this context, overexpressed Prdx3 enhances antioxidant capacity, but by inhibiting autophagy, it paradoxically contributes to increased OS, activates cell pyroptosis, and ultimately exacerbates BPH. This indicates that the enhancement in antioxidant capacity is insufficient to counterbalance the OS increase induced by other mechanisms. The aforementioned paradoxes - the coexistence of robust antioxidant expression and persistent OS - can be reconciled through the interplay of Prdx3, OS, autophagy, and pyroptosis.

Downregulating antioxidant production, such as Prdx3, can lead to ROS accumulation, which, paradoxically, may promote apoptosis under specific conditions (151). However, this ROS accumulation may not be sustained; Prdx3 downregulation can also increase mitophagy (a selective form of autophagy for mitochondria). Theoretically, ROS levels would subsequently decline due to mitophagy. However, supporting experimental evidence from subsequent studies is lacking and warrants further investigation.

Extrapolating this concept, systemically enhancing the body’s antioxidant capacity to reduce OS may inadvertently exacerbate OS. This mechanism is likely the reason why the aforementioned clinical trials failed. Indeed, reducing OS is crucial, but not all strategies targeting OS are effective.

This phenomenon aligns with findings from other research. Exercise, a strategy to enhance antioxidant capacity, increases Prdx3 expression post-exercise (152), but reduced antioxidant capacity has been observed in rats following exercise training (152). Similarly, niacin, an antioxidant-enhancing agent, was retrospectively identified as a risk factor for BPH using the NHANES database from 2003 to 2008 (153).

Therefore, within this complex network of mechanisms, it becomes evident that inhibiting Prdx3 represents a potential strategy to prevent further prostatic hyperplasia. Prdx3 inhibition maintains autophagy while reducing OS levels and suppressing cell pyroptosis. Consequently, inflammatory stimulation is mitigated or prevented, and apoptosis becomes more probable. Ultimately, by maintaining a balanced interplay between autophagy, OS, and pyroptosis, prostate hyperplasia progression can be controlled.

## Limitations and future perspectives

In this review, we delve into BPH pathogenesis and the roles of Prdx3, OS, pyroptosis, and autophagy. Existing research has flaws, like overusing preclinical models that can’t fully capture human BPH complexity. Human data on Prdx3-pyroptosis interaction is lacking, and antioxidant trials haven’t stratified patients, which hampers clinical translation.

Future research should focus on Prdx3-targeted therapy to disrupt this pathological axis. Potential strategies include: (1) Innovative Prdx3-targeted drugs like conoidin A, a potent PRDXs inhibitor (154); (2) Combining autophagy inducers (e.g., Rapamycin) with pyroptosis inhibitors (e.g., Disulfiram) (155, 156); (3) Novel approaches using selective ROS inducers to trigger apoptosis in hyperplastic cells via mitochondrial ROS production. Moreover, multi-omics integration can define BPH subtypes, and developing aged immune-dysregulation models is essential for translational validation. Despite these limitations, gaining an in - depth understanding of the complex interplay among Prdx3, OS, pyroptosis, and autophagy in BPH paves the way for developing precise treatment strategies. Future research should focus on translating these mechanistic insights into clinical applications. For instance, patient stratification analysis can be conducted to tailor personalized treatment plans based on inflammation and OS levels. This approach aims to enhance therapeutic effects and minimize side effects.

## Glossary

BPH: Benign Prostatic Hyperplasia
Prdx3: Peroxiredoxin 3
OS: Oxidative Stress
BPE Benign: Prostatic Enlargement
BPO Benign: Prostatic Obstruction
BOO: Bladder Outlet Obstruction
LUTS: Lower Urinary Tract Symptoms
IGF-1: Growth factor 1
AR: Androgen receptors
DHT: Dihydrotestosterone
VTE: Thromboembolism
E2: Estradiol
17β-HSD: 17β-hydroxysteroid dehydrogenase
SRD5A2: Steroid 5α-reductase 2
LE: Luminal epithelial cells
EMT: Epithelial-mesenchymal transition
GPER: G protein-coupled estrogen receptor ERs estrogen receptors
YAP1: Yes-associated protein 1
IR: Insulin resistance
IGF: Insulin-like growth factors
IGFBP: IGF-binding protein
THs: Thyroid hormones
PI3K: Phosphatidylinositol-3-kinase
IL-1: Interleukin-1
IL-8: Interleukin-8
TNF: Tumor necrosis factor
TRAF6: TNF receptor associated factor 6
IFN-γ: Interferon-γ
IL-17: Interleukin-17
TGF-β: Transforming growth factor-β
CXCL10: Interferon-γ-inducible protein 10
AI: Autoimmune
CMA: Chaperone-mediated autophagy
ATG: Autophagy-related genes
ER: Endoplasmic reticulum
AD: Androgen deprivation
ROS: Reactive Oxygen Species
RNS: Reactive Nitrogen Species
H2O2: Hydrogen Peroxide
BBR: Berberine
um-PEA: Palmitoylethanolamide
GZMA: Granzyme A
GSDMD: Gasdermin D
PAMP: Spathogen-associated molecular patterns
NLRs: NOD-like receptors
PYHIN: Pyrin and HIN domain-containing protein
NLRP3: NOD-like receptor thermal protein domain associated protein 3
IL-1β: Interleukin-1beta
IL-18: Interleukin-18
LPS: Lipopolysaccharides
IFN: Interferon
AMPK: Adenosine Monophosphate-Activated Protein Kinase
GlcNAc-6P: N-Acetyl-D-glucosamine 6-phosphate
TAK1: Transforming Growth Factor-β-Activated Kinase 1
GSDME-NT: N-terminal domain of GSDME
GZMB: Granzyme B
CAR T cells: Chimeric antigen receptor T cells
PRDX: Peroxiredoxins
ROS: Reactive oxygen species
H2O2: Hydrogen peroxide
NHANES: National Health and Nutrition Examination Survey
NOX: NADPH oxidase

## References

[1] Ng M Leslie SW Baradhi KM. Benign prostatic hyperplasia. In: StatPearls. StatPearls Publishing LLC., Treasure Island (FL (2025 2025). 32644346

[2] Foo KT. What is a disease? What is the disease clinical benign prostatic hyperplasia (BPH)? World J Urol. (2019) 37:1293–6. doi: 10.1007/s00345-019-02691-0, PMID: 30805683 PMC6620380

[3] Lerner LB McVary KT Barry MJ Bixler BR Dahm P Das AK. Management of lower urinary tract symptoms attributed to benign prostatic hyperplasia: AUA GUIDELINE PART I-initial work-up and medical management. J Urol. (2021) 206:806–17. doi: 10.1097/JU.0000000000002183, PMID: 34384237

[4] Ogawa T Sakakibara R Kuno S Ishizuka O Kitta T Yoshimura N. Prevalence and treatment of LUTS in patients with Parkinson disease or multiple system atrophy. Nat Rev Urol. (2017) 14:79–89. doi: 10.1038/nrurol.2016.254, PMID: 27958390

[5] Cavanaugh D Urbanucci A Mohamed NE Tewari AK Figueiro M Kyprianou N. Link between circadian rhythm and benign prostatic hyperplasia (BPH)/lower urinary tract symptoms (LUTS). Prostate. (2024) 84:417–25. doi: 10.1002/pros.24656, PMID: 38193363 PMC10922447

[6] Foo KT. Pathophysiology of clinical benign prostatic hyperplasia. Asian J Urol. (2017) 4:152–7. doi: 10.1016/j.ajur.2017.06.003, PMID: 29264224 PMC5717988

[7] Gharbieh S Reeves F Challacombe B. The prostatic middle lobe: clinical significance, presentation and management. Nat Rev Urol. (2023) 20:645–53. doi: 10.1038/s41585-023-00774-7, PMID: 37188789

[8] Santos Dias J. Benign prostatic hyperplasia: clinical manifestations and evaluation. Tech Vasc Interv Radiol. (2012) 15:265–9. doi: 10.1053/j.tvir.2012.09.007, PMID: 23244722

[9] Cannarella R Condorelli RA Barbagallo F La Vignera S Calogero AE. Endocrinology of the aging prostate: current concepts. Front Endocrinol (Lausanne). (2021) 12:554078. doi: 10.3389/fendo.2021.554078, PMID: 33692752 PMC7939072

[10] Wang H Liu C Dong Z Chen X Zhang P. Prostate-specific antigen, androgen, progesterone and oestrogen receptors in Benign prostatic hyperplasia: human tissues and animal model study. Aging Male. (2024) 27:2391380. doi: 10.1080/13685538.2024.2391380, PMID: 39140708

[11] Madersbacher S Sampson N Culig Z. Pathophysiology of benign prostatic hyperplasia and benign prostatic enlargement: A mini-review. Gerontology. (2019) 65:458–64. doi: 10.1159/000496289, PMID: 30943489

[12] Ayodele O Cabral HJ McManus D Jick S. The risk of venous thromboembolism (VTE) in men with benign prostatic hyperplasia treated with 5-alpha reductase inhibitors (5ARIs). Clin Epidemiol. (2021) 13:661–73. doi: 10.2147/CLEP.S317019, PMID: 34377032 PMC8349190

[13] Kang JW He JP Liu YN Zhang Y Song SS Xu QX. Aberrant activated Notch1 promotes prostate enlargement driven by androgen signaling via disrupting mitochondrial function in mouse. Cell Mol Life Sci. (2024) 81:155. doi: 10.1007/s00018-024-05143-0, PMID: 38538986 PMC10973062

[14] Park WY Song G Park JY Ahn KS Kwak HJ Park J. Ellagic acid improves benign prostate hyperplasia by regulating androgen signaling and STAT3. Cell Death Dis. (2022) 13:554. doi: 10.1038/s41419-022-04995-3, PMID: 35715415 PMC9205887

[15] Wang SS Li K Liu Z Gui S Liu N Liu X. Aerobic exercise ameliorates benign prostatic hyperplasia in obese mice through downregulating the AR/androgen/PI3K/AKT signaling pathway. Exp Gerontol. (2021) 143:111152. doi: 10.1016/j.exger.2020.111152, PMID: 33189835

[16] Baas W Köhler TS. Testosterone replacement therapy and BPH/LUTS. What is Evidence? Curr Urol Rep. (2016) 17:46. doi: 10.1007/s11934-016-0600-8, PMID: 27068735

[17] Saad F Yassin AA Haider A Gooren L. Effects of testosterone on the lower urinary tract go beyond the prostate: New insights, new treatment options. Arab J Urol. (2011) 9:147–52. doi: 10.1016/j.aju.2011.06.003, PMID: 26579287 PMC4150581

[18] Da Silva MHA De Souza DB. Current evidence for the involvement of sex steroid receptors and sex hormones in benign prostatic hyperplasia. Res Rep Urol. (2019) 11:1–8. doi: 10.2147/RRU.S155609, PMID: 30662879 PMC6327899

[19] Csikós E Horváth A Ács K Papp N Balázs VL Dolenc MS. Treatment of benign prostatic hyperplasia by natural drugs. Molecules. (2021) 26:7813. doi: 10.3390/molecules26237141, PMID: 34885733 PMC8659259

[20] Farnsworth WE. Estrogen in the etiopathogenesis of BPH. Prostate. (1999) 41:263–74. doi: 10.1002/(SICI)1097-0045(19991201)41:4<263::AID-PROS7>3.0.CO;2-0, PMID: 10544300

[21] Yang T Huang Y Zhou Y Chen S Wang H Hu Y. Simultaneous quantification of oestrogens and androgens in the serum of patients with benign prostatic hyperplasia by liquid chromatography-Tandem mass spectrometry. Andrologia. (2020) 52:e13611. doi: 10.1111/and.13611, PMID: 32441855

[22] Chen C Pan N Chen Z Gou C He X Wang M. The GG genotype of rs743572 in CYP17A1 gene regulating the decrease of T/E ratio can be an independent risk factor for MetS-BPH: a retrospective cohort study. World J Urol. (2024) 42:439. doi: 10.1007/s00345-024-05138-3, PMID: 39046536 PMC11269469

[23] Xue B Wu S Sharkey C Tabatabaei S Wu CL Tao Z. Obesity-associated inflammation induces androgenic to estrogenic switch in the prostate gland. Prostate Cancer Prostatic Dis. (2020) 23:465–74. doi: 10.1038/s41391-020-0208-4, PMID: 32029929 PMC7938647

[24] Sharkey C Long X Al-Faouri R Strand D Olumi AF Wang Z. Enhanced prostatic Esr1(+) luminal epithelial cells in the absence of SRD5A2. J Pathol. (2024) 263:300–14. doi: 10.1002/path.6283, PMID: 38606616 PMC11166526

[25] Fan Y Song TR Wei Q Yang L Lin T Feng XB. Modulatory effect of aquaporin 5 on estrogen-induced epithelial-mesenchymal transition in prostate epithelial cells. Chin Med J (Engl). (2020) 134:448–55. doi: 10.1097/CM9.0000000000001132, PMID: 33031138 PMC7909481

[26] De Falco M Laforgia V. Combined effects of different endocrine-disrupting chemicals (EDCs) on prostate gland. Int J Environ Res Public Health. (2021) 18:12046. doi: 10.3390/ijerph18189772, PMID: 34574693 PMC8471191

[27] Pan L Su S Li Y Liu D Shen L Wang H. The effect of acupuncture on oestrogen receptors in rats with benign prostatic hyperplasia. J Steroid Biochem Mol Biol. (2023) 234:106402. doi: 10.1016/j.jsbmb.2023.106402, PMID: 37734284

[28] McPherson SJ Hussain S Balanathan P Hedwards SL Niranjan B Grant M. Estrogen receptor-beta activated apoptosis in benign hyperplasia and cancer of the prostate is androgen independent and TNFalpha mediated. Proc Natl Acad Sci U.S.A. (2010) 107:3123–8. doi: 10.1073/pnas.0905524107, PMID: 20133657 PMC2840300

[29] Garg M Dalela D Dalela D Goel A Kumar M Gupta G. Selective estrogen receptor modulators for BPH: new factors on the ground. Prostate Cancer Prostatic Dis. (2013) 16:226–32. doi: 10.1038/pcan.2013.17, PMID: 23774084

[30] Wu WF Wang L Spetsieris N Boukovala M Efstathiou E Brössner C. Estrogen receptor β and treatment with a phytoestrogen are associated with inhibition of nuclear translocation of EGFR in the prostate. Proc Natl Acad Sci U.S.A. (2021) 118:e2103247118. doi: 10.1073/pnas.2011269118, PMID: 33771918 PMC8020780

[31] Yang T Qiu Z Shen J He Y Yin L Chen L. 17β-Estradiol, through activating the G protein-coupled estrogen receptor, exacerbates the complication of benign prostatic hyperplasia in type 2 diabetes mellitus patients by inducing prostate proliferation. J Pharm Anal. (2024) 14:100962. doi: 10.1016/j.jpha.2024.03.003, PMID: 39350964 PMC11440253

[32] Liu Z Li S Chen S Sheng J Li Z Lv T. YAP-mediated GPER signaling impedes proliferation and survival of prostate epithelium in benign prostatic hyperplasia. iScience. (2024) 27:109125. doi: 10.1016/j.isci.2024.109125, PMID: 38420594 PMC10901089

[33] Fu X Wang Y Lu Y Liu J Li H. Association between metabolic syndrome and benign prostatic hyperplasia: The underlying molecular connection. Life Sci. (2024) 358:123192. doi: 10.1016/j.lfs.2024.123192, PMID: 39488266

[34] Traish AM. Health risks associated with long-term finasteride and dutasteride use: it’s time to sound the alarm. World J Mens Health. (2020) 38:323–37. doi: 10.5534/wjmh.200012, PMID: 32202088 PMC7308241

[35] Wang Z Olumi AF. Diabetes, growth hormone-insulin-like growth factor pathways and association to benign prostatic hyperplasia. Differentiation. (2011) 82:261–71. doi: 10.1016/j.diff.2011.04.004, PMID: 21536370

[36] Jangir RN Jain GC. Diabetes mellitus induced impairment of male reproductive functions: a review. Curr Diabetes Rev. (2014) 10:147–57. doi: 10.2174/1573399810666140606111745, PMID: 24919656

[37] Yang T Zhou Y Wang H Chen S Shen M Hu Y. Insulin exacerbated high glucose-induced epithelial-mesenchymal transition in prostatic epithelial cells BPH-1 and prostate cancer cells PC-3 via MEK/ERK signaling pathway. Exp Cell Res. (2020) 394:112145. doi: 10.1016/j.yexcr.2020.112145, PMID: 32561286

[38] Miro C Di Giovanni A Murolo M Cicatiello AG Nappi A Sagliocchi S. Thyroid hormone and androgen signals mutually interplay and enhance inflammation and tumorigenic activation of tumor microenvironment in prostate cancer. Cancer Lett. (2022) 532:215581. doi: 10.1016/j.canlet.2022.215581, PMID: 35134514

[39] Lee JH Park YW Lee SW. The relationships between thyroid hormone levels and lower urinary tract symptoms/benign prostatic hyperplasia. World J Mens Health. (2019) 37:364–71. doi: 10.5534/wjmh.180084, PMID: 30644234 PMC6704305

[40] Heo JE Kim DG Yoo JW Lee KS. Metabolic syndrome-related factors as possible targets for lower urinary tract symptoms in Korean males. Aging Male. (2023) 26:6–12. doi: 10.1080/13685538.2023.2166920, PMID: 36633207

[41] Rolandi E Pescatore D Milesi GM Giberti C Sannia A Barreca T. Evaluation of LH, FSH, TSH, Prl, and GH secretion in patients suffering from prostatic neoplasms. Acta Endocrinol (Copenh). (1980) 95:23–6. doi: 10.1530/acta.0.0950023, PMID: 6779472

[42] Hata J Harigane Y Matsuoka K Akaihata H Yaginuma K Meguro S. Mechanism of androgen-independent stromal proliferation in benign prostatic hyperplasia. Int J Mol Sci. (2023) 24:11197 . doi: 10.3390/ijms241411634, PMID: 37511400 PMC10380833

[43] Du C Bhatia M Tang SC Zhang M Steiner T. Mediators of inflammation: inflammation in cancer, chronic diseases, and wound healing. Mediators Inflammation. (2015) 2015:570653. doi: 10.1155/2015/570653, PMID: 26549940 PMC4624916

[44] Gandaglia G Briganti A Gontero P Mondaini N Novara G Salonia A. The role of chronic prostatic inflammation in the pathogenesis and progression of benign prostatic hyperplasia (BPH). BJU Int. (2013) 112:432–41. doi: 10.1111/bju.12118, PMID: 23650937

[45] Bleeker J Wang ZA. Applications of vertebrate models in studying prostatitis and inflammation-associated prostatic diseases. Front Mol Biosci. (2022) 9:898871. doi: 10.3389/fmolb.2022.898871, PMID: 35865005 PMC9294738

[46] Lin D Luo C Wei P Zhang A Zhang M Wu X. YAP1 recognizes inflammatory and mechanical cues to exacerbate benign prostatic hyperplasia via promoting cell survival and fibrosis. Adv Sci (Weinh). (2024) 11:e2304274. doi: 10.1002/advs.202304274, PMID: 38050650 PMC10837380

[47] Schneider AJ Serrell EC Grimes M Wang S Bushman W. Histologic inflammation and collagen content are not positively correlated in human BPH. Prostate. (2023) 83:1529–36. doi: 10.1002/pros.24611, PMID: 37602498

[48] Devlin CM Simms MS Maitland NJ. Benign prostatic hyperplasia - what do we know? BJU Int. (2021) 127:389–99. doi: 10.1111/bju.15229, PMID: 32893964

[49] Cooper PO Yang J Wang HH Broman MM Jayasundara SM Sahoo SS. Inflammation impacts androgen receptor signaling in basal prostate stem cells through interleukin 1 receptor antagonist. Commun Biol. (2024) 7:1390. doi: 10.1038/s42003-024-07071-y, PMID: 39455902 PMC11511867

[50] Raucci F Saviano A Casillo GM Guerra-Rodriguez M Mansour AA Piccolo M. IL-17-induced inflammation modulates the mPGES-1/PPAR-γ pathway in monocytes/macrophages. Br J Pharmacol. (2022) 179:1857–73. doi: 10.1111/bph.15413, PMID: 33595097

[51] Cao D Sun R Peng L Li J Huang Y Chen Z. Immune cell proinflammatory microenvironment and androgen-related metabolic regulation during benign prostatic hyperplasia in aging. Front Immunol. (2022) 13:842008. doi: 10.3389/fimmu.2022.842008, PMID: 35386711 PMC8977548

[52] Vickman RE Aaron-Brooks L Zhang R Lanman NA Lapin B Gil V. TNF is a potential therapeutic target to suppress prostatic inflammation and hyperplasia in autoimmune disease. Nat Commun. (2022) 13:2133. doi: 10.1038/s41467-022-29719-1, PMID: 35440548 PMC9018703

[53] Oseni SO Naar C Pavlović M Asghar W Hartmann JX Fields GB. The molecular basis and clinical consequences of chronic inflammation in prostatic diseases: prostatitis, benign prostatic hyperplasia, and prostate cancer. Cancers (Basel). (2023) 15:1065. doi: 10.3390/cancers15123110, PMID: 37370720 PMC10296711

[54] Naiyila X Li J Huang Y Chen B Zhu M Li J. A novel insight into the immune-related interaction of inflammatory cytokines in benign prostatic hyperplasia. J Clin Med. (2023) 12:1038. doi: 10.3390/jcm12051821, PMID: 36902608 PMC10003138

[55] Ishiguro H Kawahara T. Nonsteroidal anti-inflammatory drugs and prostatic diseases. BioMed Res Int. (2014) 2014:436123. doi: 10.1155/2014/436123, PMID: 24900965 PMC4036408

[56] Popovics P Skalitzky KO Schroeder E Jain A Silver SV Van Fritz F. Steroid hormone imbalance drives macrophage infiltration and Spp1/osteopontin(+) foam cell differentiation in the prostate. J Pathol. (2023) 260:177–89. doi: 10.1002/path.6074, PMID: 36825524 PMC10231633

[57] Tong Y Zhou RY. Review of the roles and interaction of androgen and inflammation in benign prostatic hyperplasia. Mediators Inflammation. (2020) 2020:7958316. doi: 10.1155/2020/7958316, PMID: 33192175 PMC7641707

[58] Abo-El Fetoh ME Abdel-Fattah MM Mohamed WR Ramadan LAA Afify H. Cyclooxygenase-2 activates EGFR-ERK1/2 pathway via PGE2-mediated ADAM-17 signaling in testosterone-induced benign prostatic hyperplasia. Inflammopharmacology. (2023) 31:499–516. doi: 10.1007/s10787-022-01123-7, PMID: 36586043 PMC9958186

[59] Liu R Sun Z Wang S Liu X Man Y Chen M. Wenshenqianlie capsule improves benign prostatic hyperplasia via its anti-inflammatory and antioxidant effects. Aging (Albany NY). (2024) 16:12574–92. doi: 10.18632/aging.206103, PMID: 39237304 PMC11466478

[60] Liu Y Shao R Suo T Zhu J Liu E Wang Y. Traditional Chinese Medicine Danzhi qing’e decoction inhibits inflammation-associated prostatic hyperplasia via inactivation of ERK1/2 signal pathway. J Ethnopharmacol. (2023) 309:116354. doi: 10.1016/j.jep.2023.116354, PMID: 36906158

[61] Wang H Mu Z Liang J Li X Yang L He J. Hosta plantaginea (Lam.) Aschers flower modulates inflammation and amino acid metabolism by inhibiting NF-κB/MAPK/JAK-STAT/PI3K-Akt and AMPK pathways to alleviate benign prostatic hyperplasia in rats. J Ethnopharmacol. (2025) 337:118970. doi: 10.1016/j.jep.2024.118970, PMID: 39433163

[62] Pascal LE Dhir R Balasubramani GK Chen W Hudson CN Srivastava P. Claudin-1 down-regulation in the prostate is associated with aging and increased infiltration of inflammatory cells in BPH. Am J Clin Exp Urol. (2021) 9:53–64. doi: 10.14744/ajceu.2021.222, PMID: 33816694 PMC8012836

[63] An J Song Y Kim S Kong H Kim K. Alteration of gut microbes in benign prostatic hyperplasia model and finasteride treatment model. Int J Mol Sci. (2023) 24:4330 . doi: 10.3390/ijms24065904, PMID: 36982979 PMC10057928

[64] Li J Li Y Zhou L Li H Wan T Tang J. Microbiome analysis reveals the inducing effect of Pseudomonas on prostatic hyperplasia via activating NF-κB signalling. Virulence. (2024) 15:2313410. doi: 10.1080/21505594.2024.2313410, PMID: 38378443 PMC10880505

[65] Jain S Samal AG Das B Pradhan B Sahu N Mohapatra D. Escherichia coli, a common constituent of benign prostate hyperplasia-associated microbiota induces inflammation and DNA damage in prostate epithelial cells. Prostate. (2020) 80:1341–52. doi: 10.1002/pros.24063, PMID: 32835423

[66] Chen J Chen B Lin B Huang Y Li J Li J. The role of gut microbiota in prostate inflammation and benign prostatic hyperplasia and its therapeutic implications. Heliyon. (2024) 10:e38302. doi: 10.1016/j.heliyon.2024.e38302, PMID: 39386817 PMC11462338

[67] Dong W Zheng J Huang Y Tan H Yang S Zhang Z. Sodium butyrate treatment and fecal microbiota transplantation provide relief from ulcerative colitis-induced prostate enlargement. Front Cell Infect Microbiol. (2022) 12:1037279. doi: 10.3389/fcimb.2022.1037279, PMID: 36389141 PMC9640924

[68] Suarez Arbelaez MC Monshine J Porto JG Shah K Singh PK Roy S. The emerging role of the urinary microbiome in benign noninfectious urological conditions: an up-to-date systematic review. World J Urol. (2023) 41:2933–48. doi: 10.1007/s00345-023-04588-5, PMID: 37737900

[69] Ratajczak W Laszczyńska M Rył A Dołęgowska B Sipak O Stachowska E. Tissue immunoexpression of IL-6 and IL-18 in aging men with BPH and MetS and their relationship with lipid parameters and gut microbiota-derived short chain fatty acids. Aging (Albany NY). (2023) 15:10875–96. doi: 10.18632/aging.205091, PMID: 37847180 PMC10637784

[70] Wei P Lin D Zhang M Luo C Wu X Deng B. Cryptotanshinone modulates proliferation, apoptosis, and fibrosis through inhibiting AR and EGFR/STAT3 axis to ameliorate benign prostatic hyperplasia progression. Eur J Pharmacol. (2023) 938:175434. doi: 10.1016/j.ejphar.2022.175434, PMID: 36462735

[71] Elsherbini DMA Almohaimeed HM El-Sherbiny M Mohammedsaleh ZM Elsherbiny NM Gabr SA. Extract attenuated benign prostatic hyperplasia in rat model: effect on oxidative stress, apoptosis, and proliferation. Antioxidants (Basel) 11. (2022) 11:1094–110. doi: 10.3390/antiox11061149, PMID: 35740046 PMC9219805

[72] Wang Y Liu L Cheng C Wang S Zhai Q Song Y. Study on mechanism of Zishen Pill treating benign prostatic hyperplasia based on serum pharmacochemistry and network pharmacology. J Pharm BioMed Anal. (2023) 234:115480. doi: 10.1016/j.jpba.2023.115480, PMID: 37454501

[73] Lucas-Cava V Sánchez-Margallo FM Moreno-Lobato B Dávila-Gómez L Lima-Rodríguez JR García-Martínez V. Prostatic artery occlusion: initial findings on pathophysiological response in a canine prostate model. Transl Androl Urol. (2022) 11:1655–66. doi: 10.21037/tau-22-423, PMID: 36632152 PMC9827397

[74] Liu J Zhang J Fu X Yang S Li Y Liu J. The emerging role of cell adhesion molecules on benign prostatic hyperplasia. Int J Mol Sci 24. (2023) 24:15159. doi: 10.3390/ijms24032870, PMID: 36769190 PMC9917596

[75] Zhu C Li LY Shi MH Fang C Yang L Li T. Salmonella enterica mediated epigenetic promotion of fibrosis is a novel factor in benign prostatic hyperplasia. Mil Med Res. (2025) 12:24. doi: 10.1186/s40779-025-00614-2, PMID: 40442779 PMC12121288

[76] Cao W Li J Yang K Cao D. An overview of autophagy: Mechanism, regulation and research progress. Bull Cancer. (2021) 108:304–22. doi: 10.1016/j.bulcan.2020.11.004, PMID: 33423775

[77] Debnath J Gammoh N Ryan KM. Autophagy and autophagy-related pathways in cancer. Nat Rev Mol Cell Biol. (2023) 24:560–75. doi: 10.1038/s41580-023-00585-z, PMID: 36864290 PMC9980873

[78] Chen T Tu S Ding L Jin M Chen H Zhou H. The role of autophagy in viral infections. J BioMed Sci. (2023) 30:5. doi: 10.1186/s12929-023-00899-2, PMID: 36653801 PMC9846652

[79] Li W He P Huang Y Li YF Lu J Li M. Selective autophagy of intracellular organelles: recent research advances. Theranostics. (2021) 11:222–56. doi: 10.7150/thno.49860, PMID: 33391472 PMC7681076

[80] Klionsky DJ Petroni G Amaravadi RK Baehrecke EH Ballabio A Boya P. Autophagy in major human diseases. EMBO J. (2021) 40:e108863. doi: 10.15252/embj.2021108863, PMID: 34459017 PMC8488577

[81] Oh SH Lee DW Choi YB Lee YH Ju JS. Measurement of autophagy flux in benign prostatic hyperplasia *in vitro* . Prostate Int. (2020) 8:70–7. doi: 10.1016/j.prnil.2019.11.004, PMID: 32647643 PMC7335957

[82] Kim KH Hong GL Kim YJ Lee HJ Jung JY. Silencing of LLGL2 suppresses the estradiol-induced BPH-1 cell proliferation through the regulation of autophagy. Biomedicines. (2022) 10:1646. doi: 10.3390/biomedicines10081981, PMID: 36009528 PMC9406103

[83] Tong S Mo M Hu X Wu L Chen M Zhao C. MIR663AHG as a competitive endogenous RNA regulating TGF-β-induced epithelial proliferation and epithelial-mesenchymal transition in benign prostate hyperplasia. J Biochem Mol Toxicol. (2023) 37:e23391. doi: 10.1002/jbt.23391, PMID: 37518988

[84] Liu R Zhang S Wan R Deng J Fang W. Effect of Beclin-1 gene silencing on autophagy and apoptosis of the prostatic hyperplasia epithelial cells. Clinics (Sao Paulo). (2022) 77:100076. doi: 10.1016/j.clinsp.2022.100076, PMID: 36088885 PMC9468350

[85] Jin S Liu Z Xiang P Fu M Zhang G Li J. Activation of the cGMP/PKG/ERK signaling pathway associated with PDE5Is inhibits fibroblast activation by downregulating autophagy in early progressive benign prostatic hyperplasia. World J Urol. (2024) 42:333. doi: 10.1007/s00345-024-04956-9, PMID: 38761255

[86] Liu RF Li J Zhang J Bai PD Yang YF Li W. Crosstalk between apoptosis and autophagy in prostate epithelial cells under androgen deprivation. Exp Ther Med. (2018) 15:2263–8. doi: 10.3892/etm.2018.5726, PMID: 29456633 PMC5795527

[87] Onorati AV Dyczynski M Ojha R Amaravadi RK. Targeting autophagy in cancer. Cancer. (2018) 124:3307–18. doi: 10.1002/cncr.31335, PMID: 29671878 PMC6108917

[88] Hong GL Kim KH Kim YJ Lee HJ Kim HT Jung JY. Decreased mitophagy aggravates benign prostatic hyperplasia in aged mice through DRP1 and estrogen receptor α. Life Sci. (2022) 309:120980. doi: 10.1016/j.lfs.2022.120980, PMID: 36152678

[89] Jiang CY Yang BY Zhao S Shao SH Bei XY Shi F. Deregulation of ATG9A by impaired AR signaling induces autophagy in prostate stromal fibroblasts and promotes BPH progression. Cell Death Dis. (2018) 9:431. doi: 10.1038/s41419-018-0415-2, PMID: 29568063 PMC5864884

[90] von Zglinicki T. Oxidative stress and cell senescence as drivers of ageing: Chicken and egg. Ageing Res Rev. (2024) 102:102558. doi: 10.1016/j.arr.2024.102558, PMID: 39454760

[91] Zhao MJ Yuan S Zi H Gu JM Fang C Zeng XT. Oxidative stress links aging-associated cardiovascular diseases and prostatic diseases. Oxid Med Cell Longev. (2021) 2021:5896136. doi: 10.1155/2021/5896136, PMID: 34336107 PMC8313344

[92] Hajam YA Rani R Ganie SY Sheikh TA Javaid D Qadri SS. Oxidative stress in human pathology and aging: molecular mechanisms and perspectives. Cells. (2022) 11:552. doi: 10.3390/cells11030552, PMID: 35159361 PMC8833991

[93] Forman HJ Zhang H. Author Correction: Targeting oxidative stress in disease: promise and limitations of antioxidant therapy. Nat Rev Drug Discov. (2021) 20:652. doi: 10.1038/s41573-021-00267-5, PMID: 34257433 PMC8414784

[94] Zhang C Lan T Hou J Li J Fang R Yang Z. NOX4 promotes non-small cell lung cancer cell proliferation and metastasis through positive feedback regulation of PI3K/Akt signaling. Oncotarget. (2014) 5:4392–405. doi: 10.18632/oncotarget.2025, PMID: 24946933 PMC4147332

[95] Vital P Castro P Ittmann M. Oxidative stress promotes benign prostatic hyperplasia. Prostate. (2016) 76:58–67. doi: 10.1002/pros.23100, PMID: 26417670 PMC5469601

[96] Song G Tong J Wang Y Li Y Liao Z Fan D. Nrf2-mediated macrophage function in benign prostatic hyperplasia: Novel molecular insights and implications. BioMed Pharmacother. (2023) 167:115566. doi: 10.1016/j.biopha.2023.115566, PMID: 37778273

[97] Wang S Lu H Su M He J Tang Y Ying Y. Bisphenol H exposure disrupts Leydig cell function in adult rats via oxidative stress-mediated m6A modifications: Implications for reproductive toxicity. Ecotoxicol Environ Saf. (2024) 285:117061. doi: 10.1016/j.ecoenv.2024.117061, PMID: 39303633

[98] Li Y Liu J Liu D Wang Z Zhou Y Yang S. The prostate-associated gene 4 (PAGE4) could play a role in the development of benign prostatic hyperplasia under oxidative stress. Oxid Med Cell Longev. (2022) 2022:7041739. doi: 10.1155/2022/7041739, PMID: 35633887 PMC9135540

[99] El-Shafei NH Zaafan MA Kandil EA Sayed RH. Simvastatin ameliorates testosterone-induced prostatic hyperplasia in rats via modulating IGF-1/PI3K/AKT/FOXO signaling. Eur J Pharmacol. (2023) 950:175762. doi: 10.1016/j.ejphar.2023.175762, PMID: 37164119

[100] Abo-Youssef AM Afify H Azouz AA Abdel-Rahman HM Abdel-Naim AB Allam S. Febuxostat attenuates testosterone-induced benign prostatic hyperplasia in rats via inhibiting JAK/STAT axis. Life Sci. (2020) 260:118414. doi: 10.1016/j.lfs.2020.118414, PMID: 32926929

[101] Ihsan AU Khan FU Khongorzul P Ahmad KA Naveed M Yasmeen S. Role of oxidative stress in pathology of chronic prostatitis/chronic pelvic pain syndrome and male infertility and antioxidants function in ameliorating oxidative stress. BioMed Pharmacother. (2018) 106:714–23. doi: 10.1016/j.biopha.2018.06.139, PMID: 29990863

[102] Hussain T Tan B Yin Y Blachier F Tossou MC Rahu N. Oxidative stress and inflammation: what polyphenols can do for us? Oxid Med Cell Longev. (2016) 2016:7432797. doi: 10.1155/2016/7432797, PMID: 27738491 PMC5055983

[103] Chainy GBN Sahoo DK. Hormones and oxidative stress: an overview. Free Radic Res. (2020) 54:1–26. doi: 10.1080/10715762.2019.1702656, PMID: 31868060

[104] Jin BR Kim HJ Na JH Lee WK An HJ. Targeting benign prostate hyperplasia treatments: AR/TGF-β/NOX4 inhibition by apocynin suppresses inflammation and proliferation. J Adv Res. (2024) 57:135–47. doi: 10.1016/j.jare.2023.04.006, PMID: 37061215 PMC10918329

[105] Dos Santos L Carbone F Pacreau E Diarra S Luka M Pigat N. Cell plasticity in a mouse model of benign prostate hyperplasia drives amplification of androgen-independent epithelial cell populations sensitive to antioxidant therapy. Am J Pathol. (2024) 194:30–51. doi: 10.1016/j.ajpath.2023.09.010, PMID: 37827216

[106] Shabani E Kalantari H Kalantar M Goudarzi M Mansouri E Kalantar H. Berberine ameliorates testosterone-induced benign prostate hyperplasia in rats. BMC Complement Med Ther. (2021) 21:301. doi: 10.1186/s12906-021-03472-2, PMID: 34930229 PMC8690423

[107] D’Amico R Genovese T Cordaro M Siracusa R Gugliandolo E Peritore AF. Palmitoylethanolamide/baicalein regulates the androgen receptor signaling and NF-κB/nrf2 pathways in benign prostatic hyperplasia. Antioxidants (Basel). (2021) 10:1204. doi: 10.3390/antiox10071014, PMID: 34202665 PMC8300753

[108] Savas M Verit A Ciftci H Yeni E Aktan E Topal U. Oxidative stress in BPH. JNMA J Nepal Med Assoc. (2009) 48:41–5. doi: 10.31729/jnma.199, PMID: 19529057

[109] Vasudevan SO Behl B Rathinam VA. Pyroptosis-induced inflammation and tissue damage. Semin Immunol. (2023) 69:101781. doi: 10.1016/j.smim.2023.101781, PMID: 37352727 PMC10598759

[110] Panganiban RA Nadeau KC Lu Q. Pyroptosis, gasdermins and allergic diseases. Allergy. (2024) 79:2380–95. doi: 10.1111/all.16236, PMID: 39003568 PMC11368650

[111] Rao Z Zhu Y Yang P Chen Z Xia Y Qiao C. Pyroptosis in inflammatory diseases and cancer. Theranostics. (2022) 12:4310–29. doi: 10.7150/thno.71086, PMID: 35673561 PMC9169370

[112] Du T Gao J Li P Wang Y Qi Q Liu X. Pyroptosis, metabolism, and tumor immune microenvironment. Clin Transl Med. (2021) 11:e492. doi: 10.1002/ctm2.492, PMID: 34459122 PMC8329701

[113] Han YH Liu XD Jin MH Sun HN Kwon T. Role of NLRP3 inflammasome-mediated neuronal pyroptosis and neuroinflammation in neurodegenerative diseases. Inflammation Res. (2023) 72:1839–59. doi: 10.1007/s00011-023-01790-4, PMID: 37725102

[114] Liang H Huang Y Gao Q. Role of non-canonical pyroptosis in sepsis and other inflammatory diseases. Zhong Nan Da Xue Xue Bao Yi Xue Ban. (2021) 46:1276–84. doi: 10.12089/j.issn.1672-7347.2021.11.001, PMID: 34911863 PMC10929856

[115] Ghait M Duduskar SN Rooney M Häfner N Reng L Göhrig B. The non-canonical inflammasome activators Caspase-4 and Caspase-5 are differentially regulated during immunosuppression-associated organ damage. Front Immunol. (2023) 14:1239474. doi: 10.3389/fimmu.2023.1239474, PMID: 38106412 PMC10722270

[116] Weir A Vince JE. No longer married to inflammasome signaling: the diverse interacting pathways leading to pyroptotic cell death. Biochem J. (2022) 479:1083–102. doi: 10.1042/BCJ20210711, PMID: 35608339 PMC9162454

[117] Martinon F Mayor A Tschopp J. The inflammasomes: guardians of the body. Annu Rev Immunol. (2009) 27:229–65. doi: 10.1146/annurev.immunol.021908.132715, PMID: 19302040

[118] Lee BL Stowe IB Gupta A Kornfeld OS Roose-Girma M Anderson K. Caspase-11 auto-proteolysis is crucial for noncanonical inflammasome activation. J Exp Med. (2018) 215:2279–88. doi: 10.1084/jem.20180589, PMID: 30135078 PMC6122968

[119] Viganò E Diamond CE Spreafico R Balachander A Sobota RM Mortellaro A. Human caspase-4 and caspase-5 regulate the one-step non-canonical inflammasome activation in monocytes. Nat Commun. (2015) 6:8761. doi: 10.1038/ncomms9761, PMID: 26508369 PMC4640152

[120] Yang D He Y Muñoz-Planillo R Liu Q Núñez G. Caspase-11 requires the pannexin-1 channel and the purinergic P2X7 pore to mediate pyroptosis and endotoxic shock. Immunity. (2015) 43:923–32. doi: 10.1016/j.immuni.2015.10.009, PMID: 26572062 PMC4795157

[121] Matikainen S Nyman TA Cypryk W. Function and regulation of noncanonical caspase-4/5/11 inflammasome. J Immunol. (2020) 204:3063–9. doi: 10.4049/jimmunol.2000373, PMID: 32513874

[122] Casson CN Yu J Reyes VM Taschuk FO Yadav A Copenhaver AM. Human caspase-4 mediates noncanonical inflammasome activation against gram-negative bacterial pathogens. Proc Natl Acad Sci U.S.A. (2015) 112:6688–93. doi: 10.1073/pnas.1424371112, PMID: 25964352 PMC4450384

[123] Ai YL Wang WJ Liu FJ Fang W Chen HZ Wu LZ. Mannose antagonizes GSDME-mediated pyroptosis through AMPK activated by metabolite GlcNAc-6P. Cell Res. (2023) 33:904–22. doi: 10.1038/s41422-023-00848-6, PMID: 37460805 PMC10709431

[124] Sarhan J Liu BC Muendlein HI Li P Nilson R Tang AY. Caspase-8 induces cleavage of gasdermin D to elicit pyroptosis during Yersinia infection. Proc Natl Acad Sci U.S.A. (2018) 115:E10888–e10897. doi: 10.1073/pnas.1809548115, PMID: 30381458 PMC6243247

[125] Zheng M Karki R Vogel P Kanneganti TD. Caspase-6 is a key regulator of innate immunity, inflammasome activation, and host defense. Cell. (2020) 181:674–687.e13. doi: 10.1016/j.cell.2020.03.040, PMID: 32298652 PMC7425208

[126] Hu Y Liu Y Zong L Zhang W Liu R Xing Q. The multifaceted roles of GSDME-mediated pyroptosis in cancer: therapeutic strategies and persisting obstacles. Cell Death Dis. (2023) 14:836. doi: 10.1038/s41419-023-06382-y, PMID: 38104141 PMC10725489

[127] Liu Y Fang Y Chen X Wang Z Liang X Zhang T. Gasdermin E-mediated target cell pyroptosis by CAR T cells triggers cytokine release syndrome. Sci Immunol. (2020) 5. doi: 10.1126/sciimmunol.aax7969, PMID: 31953257

[128] Hamakawa T Sasaki S Shibata Y Imura M Kubota Y Kojima Y. Interleukin-18 may lead to benign prostatic hyperplasia via thrombospondin-1 production in prostatic smooth muscle cells. Prostate. (2014) 74:590–601. doi: 10.1002/pros.22773, PMID: 24615654

[129] Deng CH Zang ZJ Sun XZ Huang XH Wang H Wang D. Expression and significance of caspase-1 in benign hyperplastic prostate tissues. Zhonghua Nan Ke Xue. (2005) 11:810–2., PMID: 16333955

[130] Hata J Matsuoka K Harigane Y Yaginuma K Akaihata H Meguro S. Proliferative mechanism of benign prostatic hyperplasia by NLRP3 inflammasome through the complement pathway. Int J Urol. (2024) 31:1429–37. doi: 10.1111/iju.15576, PMID: 39258594

[131] Chen L Liu Y Yue S Wang H Chen J Ma W. P2X7R modulates NEK7-NLRP3 interaction to exacerbate experimental autoimmune prostatitis via GSDMD-mediated prostate epithelial cell pyroptosis. Int J Biol Sci. (2024) 20:3393–411. doi: 10.7150/ijbs.94704, PMID: 38993566 PMC11234205

[132] Matei E Enciu M Rosu MC Voinea F Mitroi AF Deacu M. Apoptosis-cell cycle-autophagy molecular mechanisms network in heterogeneous aggressive phenotype prostate hyperplasia primary cell cultures have a prognostic role. Int J Mol Sci 25. (2024) 25:7491. doi: 10.3390/ijms25179329, PMID: 39273277 PMC11394677

[133] Zang L Tian F Yao Y Chen Y Shen Y Han M. Qianliexin capsule exerts anti-inflammatory activity in chronic non-bacterial prostatitis and benign prostatic hyperplasia via NF-κB and inflammasome. J Cell Mol Med. (2021) 25:5753–68. doi: 10.1111/jcmm.16599, PMID: 33982874 PMC8184730

[134] Sun R Tian X Li Y Zhao Y Wang Z Hu Y. The m6A reader YTHDF3-mediated PRDX3 translation alleviates liver fibrosis. Redox Biol. (2022) 54:102378. doi: 10.1016/j.redox.2022.102378, PMID: 35779442 PMC9287738

[135] Hwang I Uddin MJ Lee G Jiang S Pak ES Ha H. Peroxiredoxin 3 deficiency accelerates chronic kidney injury in mice through interactions between macrophages and tubular epithelial cells. Free Radic Biol Med. (2019) 131:162–72. doi: 10.1016/j.freeradbiomed.2018.12.002, PMID: 30529270

[136] Basu A Banerjee H Rojas H Martinez SR Roy S Jia Z. Differential expression of peroxiredoxins in prostate cancer: consistent upregulation of PRDX3 and PRDX4. Prostate. (2011) 71:755–65. doi: 10.1002/pros.21292, PMID: 21031435 PMC3107902

[137] Ummanni R Barreto F Venz S Scharf C Barett C Mannsperger HA. Peroxiredoxins 3 and 4 are overexpressed in prostate cancer tissue and affect the proliferation of prostate cancer cells *in vitro* . J Proteome Res. (2012) 11:2452–66. doi: 10.1021/pr201172n, PMID: 22424448

[138] Zhao Z Cai Z Zhang S Yin X Jiang T Shen C. Activation of the FOXM1/ASF1B/PRDX3 axis confers hyperproliferative and antioxidative stress reactivity to gastric cancer. Cancer Lett. (2024) 589:216796. doi: 10.1016/j.canlet.2024.216796, PMID: 38537775

[139] Jiang MY Han ZD Li W Yue F Ye J Li B. Mitochondrion-associated protein peroxiredoxin 3 promotes benign prostatic hyperplasia through autophagy suppression and pyroptosis activation. Oncotarget. (2017) 8:80295–302. doi: 10.18632/oncotarget.17927, PMID: 29113303 PMC5655198

[140] Jiao A Sun J Sun Z Zhao Y Han T Zhang H. Effects of limonin on oxidative stress and early apoptosis in oocytes during *in vitro* maturation. Theriogenology. (2024) 218:8–15. doi: 10.1016/j.theriogenology.2024.01.025, PMID: 38290232

[141] Hong C Li X Zhang K Huang Q Li B Xin H. Novel perspectives on autophagy-oxidative stress-inflammation axis in the orchestration of adipogenesis. Front Endocrinol (Lausanne). (2024) 15:1404697. doi: 10.3389/fendo.2024.1404697, PMID: 38982993 PMC11232368

[142] Liu L McKeehan WL Wang F Xie R. MAP1S enhances autophagy to suppress tumorigenesis. Autophagy. (2012) 8:278–80. doi: 10.4161/auto.8.2.18939, PMID: 22301994 PMC3336082

[143] Li H Gao L Min J Yang Y Zhang R. Neferine suppresses autophagy-induced inflammation, oxidative stress and adipocyte differentiation in Graves’ orbitopathy. J Cell Mol Med. (2021) 25:1949–57. doi: 10.1111/jcmm.15931, PMID: 33443817 PMC7882929

[144] Xu Z Zeng X Li M Liao J Chen Q. MicroRNA-383 promotes reactive oxygen species-induced autophagy via downregulating peroxiredoxin 3 in human glioma U87 cells. Exp Ther Med. (2021) 21:439. doi: 10.3892/etm.2021.9870, PMID: 33747176 PMC7967820

[145] Xu Z Chen Q Zeng X Li M Liao J. lnc-NLC1-C inhibits migration, invasion and autophagy of glioma cells by targeting miR-383 and regulating PRDX-3 expression. Oncol Lett. (2021) 22:640. doi: 10.3892/ol.2021.12901, PMID: 34386062 PMC8299021

[146] Whitaker HC Patel D Howat WJ Warren AY Kay JD Sangan T. Peroxiredoxin-3 is overexpressed in prostate cancer and promotes cancer cell survival by protecting cells from oxidative stress. Br J Cancer. (2013) 109:983–93. doi: 10.1038/bjc.2013.396, PMID: 23880827 PMC3749568

[147] Zhao H Fu X Zhang Y Chen C Wang H. The role of pyroptosis and autophagy in the nervous system. Mol Neurobiol. (2024) 61:1271–81. doi: 10.1007/s12035-023-03614-2, PMID: 37697221 PMC10896877

[148] Cui J Zhou Z Yang H Jiao F Li N Gao Y. MST1 suppresses pancreatic cancer progression via ROS-induced pyroptosis. Mol Cancer Res. (2019) 17:1316–25. doi: 10.1158/1541-7786.MCR-18-0910, PMID: 30796177

[149] Liu J Zhou W Yang L Li Y Qiu J Fu X. STEAP4 modulates cell proliferation and oxidative stress in benign prostatic hyperplasia. Cell Signal. (2024) 113:110933. doi: 10.1016/j.cellsig.2023.110933, PMID: 37866665

[150] Luo J Mills K le Cessie S Noordam R van Heemst D. Ageing, age-related diseases and oxidative stress: What to do next? Ageing Res Rev. (2020) 57:100982. doi: 10.1016/j.arr.2020.100982, PMID: 31733333

[151] Zhao Z Sun Y Tang J Yang Y Xu X. LRPPRC regulates Malignant behaviors, protects mitochondrial homeostasis, mitochondrial function in osteosarcoma and derived cancer stem-like cells. BMC Cancer. (2023) 23:935. doi: 10.1186/s12885-023-11443-8, PMID: 37789316 PMC10548780

[152] Kwon I Song W Jang Y Choi MD Vinci DM Lee Y. Elevation of hepatic autophagy and antioxidative capacity by endurance exercise is associated with suppression of apoptosis in mice. Ann Hepatol. (2020) 19:69–78. doi: 10.1016/j.aohep.2019.08.010, PMID: 31611063

[153] Feng X Chen Y Xia W Zhang B. Association between dietary niacin intake and benign prostatic hyperplasia: a population-based results from NHANES 2003-2008. J Health Popul Nutr. (2024) 43:130. doi: 10.1186/s41043-024-00624-1, PMID: 39174993 PMC11342560

[154] Ryu DY Kim KU Kwon WS Rahman MS Khatun A Pang MG. Peroxiredoxin activity is a major landmark of male fertility. Sci Rep. (2017) 7:17174. doi: 10.1038/s41598-017-17488-7, PMID: 29215052 PMC5719347

[155] Yu WX Lu C Wang B Ren XY Xu K. Effects of rapamycin on osteosarcoma cell proliferation and apoptosis by inducing autophagy. Eur Rev Med Pharmacol Sci. (2020) 24:915–21. doi: 10.26353/eurrev.v24i1.16, PMID: 32016998

[156] Hu JJ Liu X Xia S Zhang Z Zhang Y Zhao J. FDA-approved disulfiram inhibits pyroptosis by blocking gasdermin D pore formation. Nat Immunol. (2020) 21:736–45. doi: 10.1038/s41590-020-0669-6, PMID: 32367036 PMC7316630

